# An adaptive visual diagnostic model for complex scenes based on unsupervised pseudo-label continuous learning and BiFPN-enhanced YOLO

**DOI:** 10.3389/fnbot.2026.1911412

**Published:** 2026-08-27

**Authors:** Xingque Xu, Lifei Li, Shaoyan Jiang, Yongjian Li, Bei Li

**Affiliations:** Guangdong Power Grid Co., Ltd. Zhongshan Power Supply Bureau, Zhongshan, Guangdong, China

**Keywords:** bidirectional feature pyramid network, continuous learning, dynamic pseudo-labels, forgetting degradation, unsupervised domain adaptation, visual diagnosis

## Abstract

With the deployment of intelligent systems in open environments, visual perception capabilities are required for engineering applications. Traditional static visual detection models experience feature degradation under environmental changes. Conventional pseudo-labeling techniques exhibit confirmation bias when processing complex samples, and models encounter knowledge forgetting during the continuous absorption of unlabeled data streams. This paper constructs an adaptive visual diagnostic framework for complex scenes, integrating unsupervised domain adaptation with continuous learning. At the feature extraction level, by introducing a BiFPN node, the network compensates for multi-scale spatial distortions caused by environmental changes while maintaining computational economy. A pseudo-label bidirectional recovery mechanism constrained by dynamic thresholds is utilized to extract supervision signals and alleviate confirmation bias. During the parameter iteration phase, a structured predictive distillation loss function is integrated to mitigate the knowledge forgetting phenomenon during continuous data absorption. Tests on the BDD100K dataset show that, with a computational load of 16.6 GFLOPs and an inference speed of 19.3 FPS, the model records an mAP@0.5 of 43.5% and 47.1%, and an mAP@0.5:0.95 of 22.1% and 24.5% in the illumination and meteorological domain transfer tasks, respectively. In the Cityscapes fog transfer test, the model reaches an mAP@0.5 of 48.3% and an mAP@0.5:0.95 of 25.1%. After 10 rounds of incremental evolution, the source domain mAP@0.5 remains at 52.8%, demonstrating knowledge retention capabilities comparable to baseline continuous learning methods.

## 1 Introduction

Visual diagnostics and environmental perception models are core components for ensuring the safety decisions of complex dynamic systems (such as smart city nodes, industrial automation and high-precision monitoring equipment) (Pandharipande et al., 2023). The visual diagnostic framework discussed in this paper breaks through the limitations of traditional medical and industrial defect detection, aiming to evaluate and repair the feature representation degradation problem of complex dynamic systems (taking the autonomous driving perception domain as an example) when facing non-stationary environmental inputs such as meteorological changes and light distortion. Layered perception enhancement technology requires the model to provide stable and high-precision feature mapping in extremely variable open environment layers (Zha et al., 2024). The YOLO series of algorithms have dominated many general visual diagnostic tasks due to their end-to-end prediction mechanism and efficient inference (Hussain, 2024). Among these variants, the YOLOv5 architecture provides a highly decoupled gradient optimization path and a stable parameter space. Newer detection models (such as YOLOv8 or YOLOv10) achieve higher accuracy on closed-set datasets; their complex dual-label assignment strategies or anchor-free dense prediction mechanisms are prone to inducing gradient conflicts during backpropagation when facing continuous distribution shifts in unsupervised target domains. The rationale for selecting YOLOv5s as the baseline framework lies in its lightweight structural balance, which effectively supports long-cycle continuous learning iterations on edge devices and restricts knowledge forgetting caused by drastic parameter drifts. The development of edge deep learning technology has further promoted the migration and deployment of computer vision and medical, remote sensing and other diagnostic models to mobile terminals and resource-constrained devices (Xu et al., 2025). In real open environments, the drastic fluctuations in ambient light, severe weather conditions (such as rain, snow and fog) and severe overlapping and occlusion of targets make it extremely difficult to obtain stable and high-quality visual features (Dasu et al., 2026). The data distribution in such complex scenarios exhibits high non-stationarity and unpredictability (Sridevi and Harish, 2024). When faced with such spatial and temporal distribution shifts (Domain Shift), traditional static visual detection models often overfit their underlying convolutional kernels to specific texture and photometric features of the source domain. When the physical rendering conditions of the test domain change abruptly, the model is prone to feature extraction bottlenecks, resulting in a significant increase in the false negative and false positive rates of small targets or blurred edge features (Liu Z. et al., 2025).

To cope with the background noise and multi-scale interference brought about by complex scenes, existing studies have focused on building lightweight resource perception frameworks or using feature aggregation to strengthen the response weights of target regions (Shiddiqi et al., 2025). Introducing deformable convolution and feature alignment structures can help alleviate the spatial deformation and feature divergence problems in multi-scale target detection (Fu et al., 2023). In terms of cross-scale spatial element processing, the Bi-FPN-YOLO network has shown good deep and shallow semantic fusion capabilities and computational economy (Doherty et al., 2025). Most of these improved architectures rely on closed and independent datasets for supervised training, and their underlying logic strictly assumes that the test data and training data follow independent and identical distributions. The visual diagnosis scene in the real world is continuous, open and dynamically evolving, and the deduction of the physical environment will lead to continuous domain changes in the input data stream. When the pre-trained visual diagnosis model is deployed to a new illumination domain or meteorological domain, the lack of accurate supervision signals of manually labeled data in the target domain makes it difficult for the underlying perception network to spontaneously complete cross-domain alignment of feature space. The traditional offline retraining mode not only faces the high cost of massive data collection and manual labeling, but also cannot meet the real-time incremental perception requirements of edge dynamic systems for continuous data streams.

Unsupervised domain adaptation constructs a crucial theoretical pathway for the scalable deployment of visual diagnostic systems in open environments. In practical engineering deployments, self-supervised learning using pseudo-labels serves as a common strategy for feature space reconstruction. Traditional fixed-threshold pseudo-labeling techniques, when processing complex edge samples, easily misinterpret high-confidence background noise as foreground targets, leading to severe confirmation bias and distorting feature decision boundaries. Simultaneously, as the model continuously absorbs new domain data streams, the underlying representation logic is prone to shifting towards local optima in the new distribution, increasing the risk of forgetting historical perceptual memory. To overcome the paradox of feature degradation and knowledge forgetting in open environments, this paper constructs a cascaded adaptive perception computational framework. This framework systematically integrates cross-scale feature enhancement, dynamic pseudo-label evolution, and continuous learning. Traditional feature pyramids (such as PANet) experience equal-weight information decay when processing cross-scale semantics, and alternative Transformer-based attention mechanisms introduce substantial computational overhead. An optimized BiFPN fusion node is introduced at the feature extraction end, utilizing its adaptive-weight skip connections to compensate for multi-scale spatial distortions caused by environmental changes while maintaining computational economy. Conventional fixed-threshold pseudo-labeling techniques induce severe confirmation bias when processing complex edge samples. This study employs a dynamic threshold constraint and a bidirectional spatial recovery mechanism, replacing singular static filtering logic, to extract high-purity supervision signals from unlabeled data streams. At the self-supervised evolution end, joint constraint boundaries of dynamic thresholds and bidirectional spatial recovery are established to suppress noise accumulation in the target domain’s unlabeled data stream. At the parameter iteration end, an incremental continuous learning mechanism is established combined with predictive distillation. This collaborative architecture aims to enhance generalization perception in new domains while suppressing the degradation of historical diagnostic knowledge from a parameter optimization perspective, providing a viable engineering path for long-term visual diagnosis on edge computing nodes.

## 2 Related work

Starting with the phenomenon of visual feature degradation at night due to drastic fluctuations in illumination, existing studies have revealed the interference of low illumination and complex background noise on the geometric contours and high-frequency texture resolution of targets (Peng and Jiang, 2026). In the case of barriers between source domain data and target environment distribution, cross-domain adaptive target detection has begun to establish a robust mapping bridge in dynamic open environments through refined knowledge transfer and implicit feature space guidance (Wang et al., 2023). In view of the temporal non-stationarity in complex visual diagnostic tasks, the network architecture combining YOLO and CNN-BiGRU can efficiently capture spatiotemporal sequence dependencies, which has been verified in infrastructure anomaly assessment (Zhumadillayeva et al., 2025). Traditional feature pyramids are often accompanied by information decay when dealing with cross-resolution semantic transmission. Multi-scale feature weighted fusion mechanism has been widely introduced into multi-task perception models, improving the scene resolution accuracy and diagnostic granularity in panoramic visual environments (Niu and Zhang, 2025). Deeply integrating the attention mechanism with the bidirectional feature pyramid (BiFPN) breaks the limitation of unidirectional information flow and enhances the sensitivity of deep learning models to small and fuzzy features (Li et al., 2022). Multi-scale multi-view learning networks, with their structure-aware characteristics, are suitable for autonomous driving scenarios, and also demonstrate stable cross-domain generalization and feature extraction capabilities in medical visual diagnosis tasks such as fundus multi-disease classification (Xie et al., 2025). In the face of roadside scenarios with wide data distribution or overlapping target spaces, the optimized network based on multi-scale feature pyramids improves the recall rate of edge diagnosis samples through cross-layer semantic alignment (Zheng et al., 2025).

As visual diagnostics are increasingly deployed to terminal devices, the optimized design of lightweight neural networks provides underlying computing power for real-time perception on edge computing devices through channel pruning and depthwise separable convolution (Liu and Xu, 2025). For complex scenarios such as SAR radar images containing electromagnetic interference, multi-scale feature fusion and dynamic alignment mechanisms show that optimization at the network structure level helps improve the robustness of the model to background noise (Liu S. et al., 2025). An edge deployment framework for safety-critical tasks balances diagnostic accuracy and inference latency under resource-constrained hardware conditions by constructing a multi-scale and multi-target parallel detection architecture (Zhu et al., 2026). The multi-layer fine-grained feature extraction strategy deeply explores the high-resolution spatial coordinates of shallow networks, providing theoretical support for the model to capture minute anomaly details (Zuo et al., 2024). In the detection of high-frequency information such as traffic signs, the single-stage algorithm with attention enhancement suppresses the transmission of redundant background information by generating spatial masks (Huang et al., 2022). The evolution of visual positioning technology has enabled perception models to make new progress in understanding the interaction relationships between entities in complex scene contexts (Xiao et al., 2025). Anchor-free multi-task learning networks eliminate the hard scale matching error and hyperparameter dependence caused by preset prior anchor boxes in panoramic perception tasks by predicting pixels one by one (Zhan et al., 2024). By simplifying the YOLOv5 network architecture to adapt to the deployment of real crop monitoring scenarios, the engineering applicability of lightweight detection models in outdoor unstructured and highly dynamic environments has been verified (Nnadozie et al., 2024). The real-time end-to-end target detection framework YOLOv10 circumvented the non-maximum suppression post-processing stage by introducing a dual-label allocation strategy, optimizing the overall latency of the inference pipeline and the system (Wang A. et al., 2024). The local and global feature aggregation mechanism strengthens the spatial logical correlation between isolated target entities and large-scale environmental backgrounds in remote sensing image detection (Zhang Y. et al., 2025). The application of deep learning in the detection of tiny defects in electronic components further proves the deep analytical ability of modern detection models for high-density and complex micro-textures (Montoya Magaña et al., 2025). The lightweight target detector based on feature splicing and cross-level fusion expands the application boundary of edge vision sensors in multi-task concurrent scenarios by relying on extremely low parameter redundancy (Xue et al., 2024).

In visual perception in open environments, unsupervised learning and pseudo-label optimization mechanisms are key ways to overcome the bottleneck of massive manual annotation costs in target domains (Li et al., 2023). By dynamically generating prediction results of the target domain under weak or unsupervised conditions as supervision signals, the model can autonomously extract potential distribution patterns in complex natural scene images (Wang et al., 2021). The confidence-aware query label strategy in the testing phase improves the generalization performance of 3D small sample segmentation networks in unknown distribution domains by evaluating prediction certainty online (Mozafari et al., 2025). The use of high-quality synthetic labels in light field unsupervised target detection for knowledge guidance has been proven to replace a large number of real pixel-level annotations in the directional update of network gradients (Zheng et al., 2024). Traditional pseudo-labels are prone to misleading in low confidence regions, inducing serious confirmation bias. In semi-supervised multimodal tasks, the two-stage knowledge distillation network with multi-teacher self-training reduces the label confirmation bias caused by the limited field of vision of a single teacher model through multi-source cross-validation logic (Wu et al., 2025). In the diagnosis of degradation faults under limited data conditions, pseudo-label-assisted semi-supervised adversarial reinforcement learning successfully reconstructed the smooth classification boundary of high-dimensional features in the latent space (Chen et al., 2025).

To reduce the domain shift in different environments, the semi-supervised domain adaptive YOLO model alleviates the data distribution divergence difference in cross-domain object detection by generating adversarial training and feature-level edge alignment (Zhou et al., 2023). The attention-based single-stage detector domain adaptation method forces the model’s receptive field to focus on the core target shape that is domain invariant, thereby filtering out most of the domain-specific background noise at the front end (Vidit and Salzmann, 2022). The collaborative mechanism of self-training and adversarial learning provides a smoother source domain to target domain feature transition constraint for domain adaptive object detection by jointly optimizing the target domain distribution difference and task classification loss (Munir et al., 2021). The uncertainty perception mechanism has been proven to be crucial in the unsupervised domain adaptation process. By strictly evaluating the logical information entropy of the network prediction results, it blocks the back propagation of high-risk edge noise samples and reduces the accumulation of erroneous gradients (Guan et al., 2021). This domain-adaptive object detection strategy has been widely transferred to extreme weather scenarios such as fog and rain to mitigate the visual feature distortion caused by specific weather scattering models (Li et al., 2024). The mutual learning framework, combined with the dynamic screening of highly reliable pseudo-labels, breaks the vicious cycle of self-reinforcement of erroneous labels in semi-supervised medical image segmentation (Su et al., 2024). The application of large-scale pre-trained visual models in removing interference from severe weather reveals the direct benefit of sufficient prior knowledge base to low-level image restoration and high-level semantic maintenance (Tan et al., 2024). In the highly concealed camouflaged target detection task, the uncertainty-aware collaborative mechanism greatly improves the pixel-level discrimination ability of the model in low-contrast and high-noise environments (Yang et al., 2026). Recent advancements in unsupervised domain adaptation have gradually expanded towards multi-source distributions and source-free transfer scenarios. Causal-guided adaptive multimodal diffusion networks mitigate distribution discrepancies by aligning underlying causal representations across multiple source domains (Cai et al., 2025). Concurrently, attention cycle-consistent universal networks broaden the processing scope of category shift, improving feature alignment efficiency in universal domain adaptation tasks (Cai et al., 2024). In scenarios lacking source data, dynamic uncertainty-driven confident fusion strategies reconstruct reliable target domain distributions through multi-source-free adaptation, providing a novel perspective for self-supervised knowledge evolution (Cai et al., 2026). The above-mentioned domain adaptation methods are mostly limited to static migration from a single source domain to a single target domain. Under continuous dynamic data flow input, the model is prone to falling into the dilemma of stability and plasticity. Continuous learning theory provides a systematic mathematical discourse to deal with catastrophic forgetting (Wang L. et al., 2024). Combined with the incremental learning paradigm of small sample classes, the diagnostic model can effectively solidify the memory of the basic features of the old domain while absorbing the knowledge flow of the new domain (Zhang J. et al., 2025). The decoupled domain knowledge-assisted learning architecture provides a rigorous mathematical basis for separating new and old knowledge representations for continuous target detection in open domains. By constraining the offset trajectory of the parameter space, it provides a novel solution to alleviate forgetting in the incremental learning process (He et al., 2025).

## 3 Methodology

**Figure 1:**
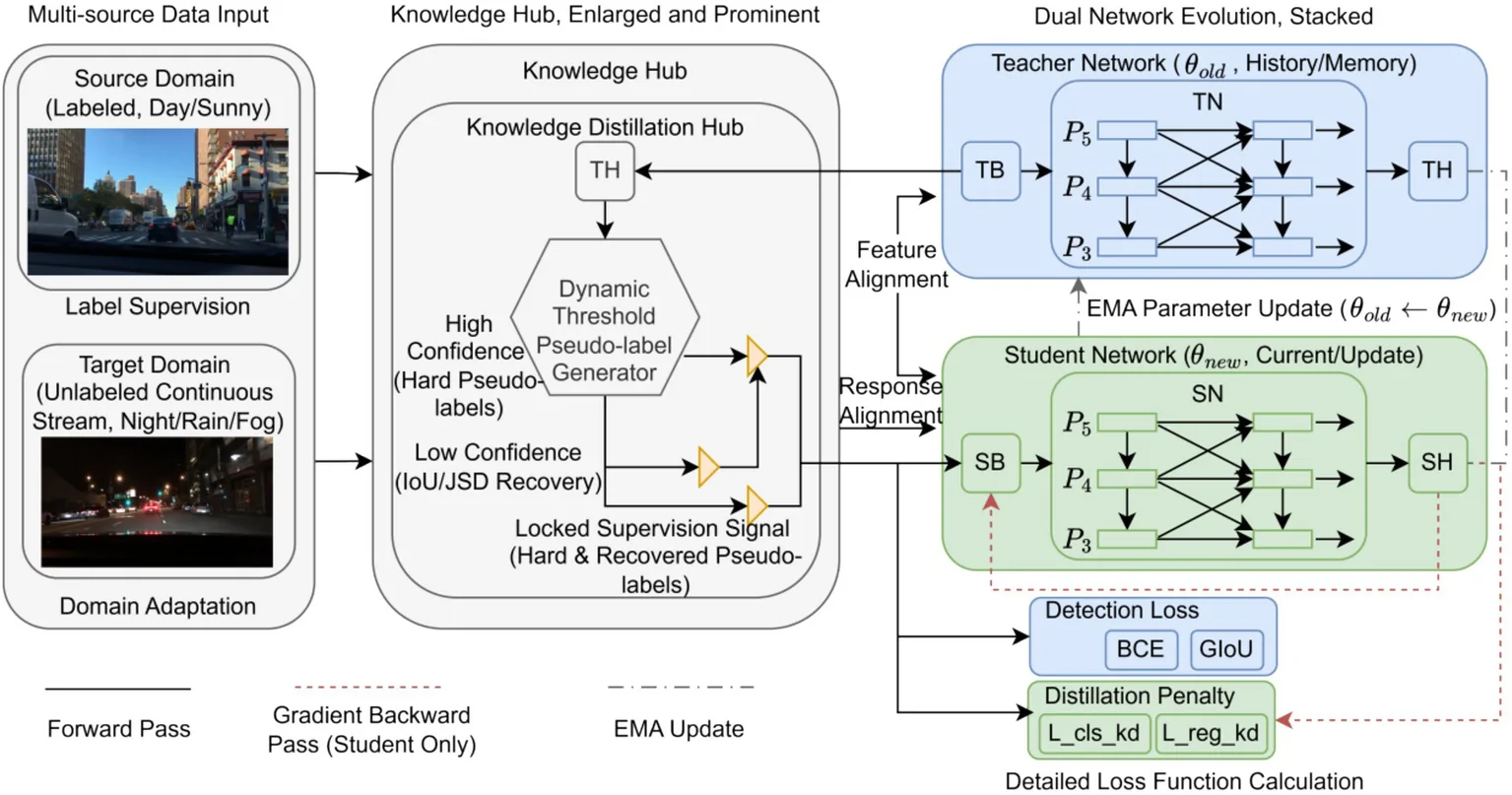
Overall architecture of the dual-network visual diagnostic system for cross-domain transfer, integrating dynamic pseudo-label generation and predictive distillation.

When deployed in complex and non-stationary environments, visual diagnostic systems have long faced three core challenges: multi-scale feature distortion, missing supervision signals in the target domain, and historical knowledge forgetting driven by continuous data streams. Traditional single-stage detection algorithms, constrained by static feature pyramids and closed-set distribution assumptions, are prone to severe degradation risks in feature alignment when faced with domain shifts caused by sudden weather changes and rapid fluctuations in light intensity. Overcoming the theoretical bottlenecks of cross-domain robust perception and continuous model evolution relies on the tight coupling of multi-level feature enhancement and knowledge protection mechanisms. In the adaptive global architecture constructed in this section, the components do not operate independently but form an end-to-end optimization closed loop: the high-quality feature responses provided by BiFPN serve as the foundation for dynamic threshold pseudo-label generation; the high-purity pseudo-labels obtained through bidirectional recovery further provide reliable supervision signals for the predictive distillation of the teacher-student network. This integration strategy helps mitigate the gradient conflicts often seen in traditional isolated modules when dealing with continuous, non-stationary data streams. Its multi-dimensional perception and parameter optimization topology is shown in Figure 1.

As shown in the global flow logic of Figure 1, this adaptive perception system consists of three parallel closed loops: cross-domain data alignment, self-supervised signal mining, and asymmetric knowledge transfer. At the feature abstraction level, a deeply optimized BiFPN multi-scale fusion topology is embedded synchronously in the neck of the Teacher Network and Student Network to strengthen the interactive association of cross-resolution semantics and the correction of physical spatial distortion. When facing target domains that have completely lost manual annotation (such as continuously input nighttime or rainy/foggy image streams), the system constructs an independently operating Knowledge Hub. It relies on a Dynamic Threshold Pseudo-label Generator and a low-confidence bidirectional spatial recovery engine (IoU/JSD Recovery) to strip away background noise online, providing high-purity hard pseudo-label supervision for network iteration. In the incremental evolution dimension, the architecture relies on the teacher model with frozen source domain feature mappings to constrain the real-time updated student model, and rigorously decouples the optimization objective of the gradient space into a pseudo-label-driven detection loss and a distillation penalty term aimed at suppressing knowledge forgetting. The following section will delve into the underlying tensor network of this computational framework, and will provide a detailed breakdown of the multi-scale feature fusion mechanism, the dynamic pseudo-label generation constraint boundary, and the mathematical modeling and joint optimization conditions of the domain incremental continuous learning system.

### 3.1 Adaptive fusion of multi-scale features enhanced by BiFPN

The path aggregation network (PANet) used in the traditional YOLO architecture transmits features through bidirectional paths from bottom to top and from top to bottom. This equal-weight node connection method ignores the difference in the semantic contribution of feature maps with different resolutions in complex scenes. Small-scale targets are prone to losing their spatial location information in deep feature maps after multiple downsampling operations, while large targets are easily interfered with by shallow high-frequency noise. Among alternative solutions for multi-scale feature aggregation, Adaptively Spatial Feature Fusion (ASFF) or Transformer-based fusion mechanisms can improve global perception, but they exponentially increase the memory footprint and inference latency on edge devices. By removing redundant edges of single-input nodes and introducing cross-scale skip connections with adaptive weights, BiFPN achieves a better balance between feature representation capability and computational complexity. Studies on adaptive multi-scale image fusion and contrast optimization have shown that attention-enhanced BiFPN can optimize the alignment efficiency of cross-scale distortion features (Zhang and Han, 2026). To alleviate spatial deformation and feature inaccuracy of multi-scale diagnostic targets, the native YOLO feature fusion neck is replaced with a Bidirectional Feature Pyramid Network (BiFPN).

**Figure 2:**
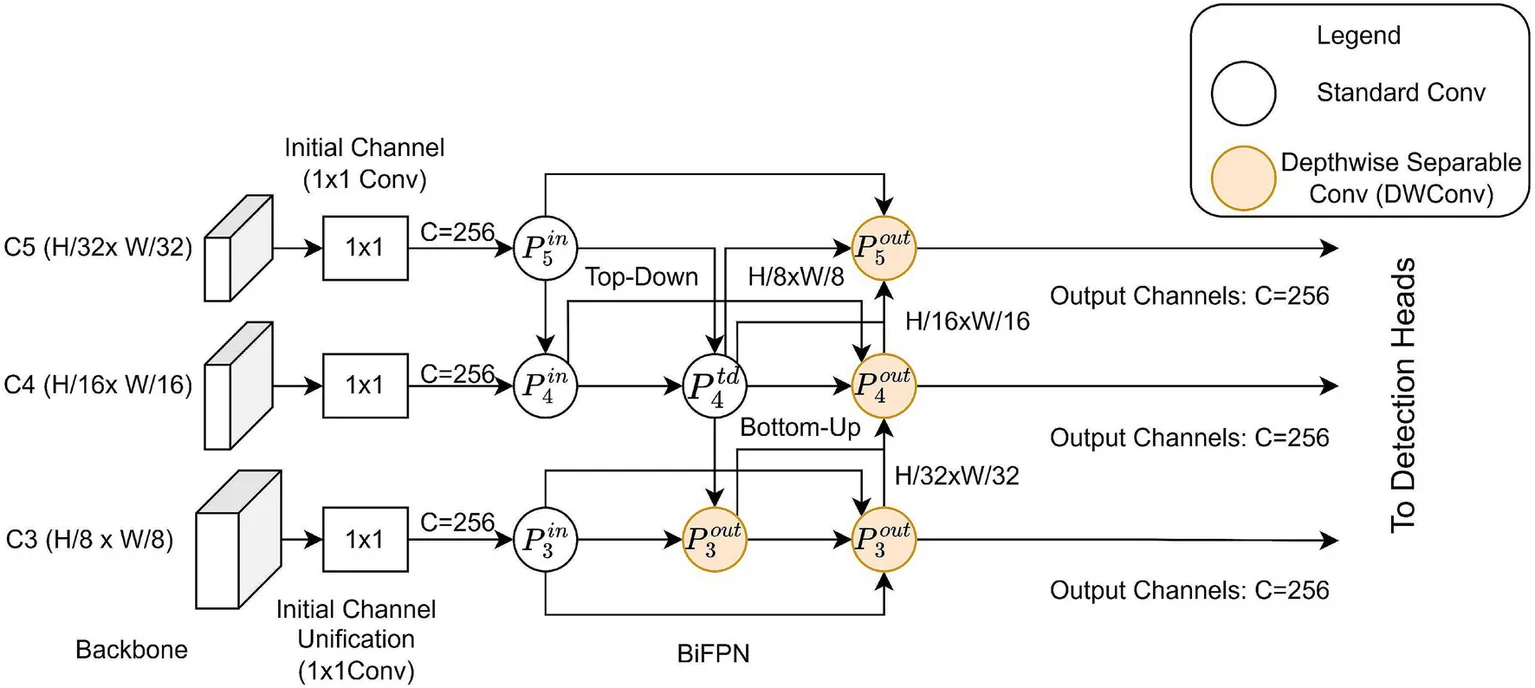
Topological structure of the modified multi-scale feature fusion neck utilizing BiFPN and target Depthwise Separable Convolution (DWConv) substitution.

The modified Neck topological structure and feature flow are illustrated in Figure 2. In the forward propagation of the tensor dimension, the input image extracted by the backbone network outputs three feature layers {C3,,C4,,C5} with spatial downsampling rates of 8x, 16x, and 32x. The corresponding input spatial resolutions are H/8×W/8 , H/16×W/16 , and H/32×W/32 . In the standard visual detection framework, very shallow features contain background redundant noise, and their extremely high spatial resolution will cause an increase in computational overhead. Due to the balance between high-level semantic abstraction and terminal computing power constraints, the model discards the sum layers and extracts the aforementioned feature layers, using a 1×1 convolution to uniformly compress their channel number to 256 to form the core pyramid. BiFPN constructs a computationally efficient topology by removing redundant nodes on single input edges and establishing direct skip connections between input and output nodes at the same level. To break the limitation of equal-weighted fusion of features at different levels, the model adopts a Fast Normalized Fusion mechanism to dynamically calculate the contribution of input nodes. The output feature $\;P_{i}^{\text{out}}\;$ of the level is defined as the weighted sum of the features of all associated input nodes:

$$P_{i}^{\text{out}}=\sum_{j}\frac{w_{j}}{\in_{1}+\sum_{k}w_{k}}\cdot \Phi (P_{j}^{\text{in}})$$ (1)

In Equation 1, $w_{j}$ represents the non-negative scalar weights dynamically learned by the network during training. Strict non-negativity is ensured by appending a ReLU activation function to the weight variables. The parameter $\in_{1}$ is set $1\times 10^{-4}$ to ensure the numerical stability of the underlying tensor operations and effectively mitigate the risk of division-by-zero overflow. In a half-precision floating-point (FP16) training and inference architecture deployed for edge devices, this constant is not only much higher than the underflow threshold of FP16, but also ensures smooth convergence of the network in extreme parameter states (such as multi-channel weights approaching zero) without changing the physical premise of the relative distribution of node input weights. Φ(·) represents the feature resolution alignment operation (including downsampling max pooling or upsampling bilinear interpolation).

Because multi-scale feature networks have upper and lower topological boundaries, the node fusion paths at different semantic depths exhibit asymmetry. Taking the $\left\{P_{3},,P_{4},,P_{5}\right\}$ constructed pyramid hierarchy as an example, $P_{3}$ the forward propagation tensor flow directions of the bottom-level nodes, intermediate nodes $P_{4}$ , and top-level nodes are rigorously defined as follows $P_{5.}$

The bottom-level node, $P_{3}$ as the highest-resolution shallow feature, only receives top-down information from the original path and intermediate layers, as defined in Equation 2:

$$P_{3}^{\text{out}}=\text{Conv}(\frac{w_{1}\cdot P_{3}^{\text{in}}+w_{2}\cdot \text{Resize}(P_{4}^{\mathrm{td}})}{w_{1}+w_{2}+\in_{1}})$$ (2)

Intermediate nodes $P_{4}$ possess complete cross-scale interaction channels, requiring the maintenance of intermediate state tensors $P_{4}^{\mathrm{td}}$ and final output state tensors $P_{4}^{\text{out}}$ . The flow operations of these two tensors constitute the joint mechanism defined in Equation 3:

$$\begin{array}{c} \left\{ \begin{array}{rr} P_{4}^{\mathrm{td}} & =\text{Conv}(\frac{w_{1}\cdot P_{4}^{\text{in}}+w_{2}\cdot \text{Resize}(P_{5}^{\text{in}})}{w_{1}+w_{2}+\in_{1}})\\ P_{4}^{\text{out}} & =\text{Conv}(\frac{{w_{1}}^{\prime }\cdot P_{4}^{\text{in}}+{w_{2}}^{\prime }\cdot P_{4}^{\mathrm{td}}+{w_{3}}^{\prime }\cdot \text{Resize}(P_{3}^{\text{out}})}{{w_{1}}^{\prime }+{w_{2}}^{\prime }+{w_{3}}^{\prime }+\in_{1}}) \end{array}\right. \end{array}$$ (3)

The top-level node $P_{5}$ contains abstract high-level global semantics. Its fusion skips the top-down intermediate transformations and directly aggregates the original input and the features passed down from the lower levels, as defined in Equation 4:

$$\begin{array}{c} P_{5}^{\text{out}}=\text{Conv}(\frac{w_{1}\cdot P_{5}^{\text{in}}+w_{2}\cdot \text{Resize}(P_{4}^{\text{out}})}{w_{1}+w_{2}+\in_{1}}) \end{array}$$ (4)

In the above multi-level topology propagation formula, $P_{n}^{\text{in}}$ represents the initial feature tensor passed from the corresponding level of the backbone network; $P_{n}^{\mathrm{td}}$ represents the intermediate transitional features generated in the top-down feature flow path; $P_{n}^{\text{out}}$ and represents the multi-scale fusion features finally output to the detector head. Resize(·) refers to the spatial resolution dynamic matching operation, which performs upsampling (bilinear interpolation) when receiving high-level features and downsampling (max pooling) when receiving low-level features. The variables $w_{1}$ and $w_{2}$ denote the learnable scalar weights for the top-down intermediate fusion pathways. The variables ${w_{1}}^{\prime }$ , ${w_{2}}^{\prime }$ , and $\;{w_{3}}^{\prime }\;$ represent the independent learnable weights for the bottom-up output pathways, corresponding to the original input $\;P_{4}^{\text{in}}\;$ , the intermediate transitional tensor $\;P_{4}^{\mathrm{td}}$ , and the lower-level features $P_{3}^{\text{out}}$ , respectively. These two sets of weights are updated independently during backpropagation, enabling the network to adaptively calibrate the semantic contributions from different spatial flow directions at distinct feature aggregation stages.

Within the multi-scale feature interaction of BiFPN, the convolution operations at the specific output nodes ( $P_{3}^{\text{out}}$ , $P_{4}^{\text{out}}$ , and $\;P_{5}^{\text{out}}$ , as marked in Figure 2) are configured as Depthwise Separable Convolutions (DWConv). This configuration maintains cross-scale semantic fusion capabilities while decoupling spatial and channel features, thereby reducing the inference parameter count of the model. Other input and intermediate transition nodes retain standard convolution operations. During backpropagation, the model dynamically adjusts the relative size of the weight matrices of each layer node based on the loss gradient returned by the detection head. When the target is subject to overlapping interference or partial occlusion in complex scenes, the network reduces the fusion weight of the contaminated pixel level through this differentiated boundary fusion formula, increasing the fusion ratio of global high-dimensional semantics or local edge features to reconstruct the spatial geometric contour of the target.

### 3.2 Dynamic threshold-driven unsupervised pseudo-label generation and bidirectional recovery

The fusion of feature pyramids is based on high-quality gradient backpropagation. When a visual diagnostic model is deployed to a new test domain (e.g., from daytime to rainy night), it loses real human label supervision. The self-supervised paradigm based on bidirectional pseudo-label recovery has shown effective confidence verification ability in cross-domain object detection (Sajid et al., 2024). Using source domain pre-trained models to predict target domain data to generate pseudo-labels is the mainstream path to achieve unsupervised adaptation. In the classic semi-supervised learning mechanism, a basic decision probability boundary of 0.5 or a high confidence prior value of 0.7 is usually used as a fixed global threshold to filter pseudo-labels. This static global constraint causes class bias: a high threshold will miss difficult samples, and a low threshold will induce the network to fit the wrong background.

In unsupervised anomaly detection, the adaptive threshold mechanism can dynamically calibrate the confidence boundary of the model based on the temporal distribution characteristics of the data (Ding et al., 2026). Existing adaptive thresholding methods in semi-supervised learning typically adjust confidence boundaries based on learning status or moving averages. FlexMatch scales thresholds utilizing class-specific learning difficulties (Zhang et al., 2021). FreeMatch maintains an exponential moving average of global and local confidence predictions (Mao et al., 2023). Dash dynamically adjusts the threshold curve based on the training loss (Deng et al., 2022). The dynamic threshold mechanism proposed in this module introduces a standard deviation penalty margin ( $\sigma_{t}^{c}$ ) alongside the temporal moving average ( $\mu_{t}^{c}$ ). This dual-constraint approach explicitly captures the fluctuation variance of non-stationary data streams in open environments. Standard methods tracking only mean confidence often experience bias when environmental shifts cause abrupt spikes in data variance. The inclusion of the standard deviation margin dynamically truncates the confidence probability space, ensuring stable pseudo-label generation under severe domain shifts. The module designs a dynamic threshold generator that is constrained by both the training iteration time and the class statistical distribution. Let t the model output a set of predicted boxes $B_{t}={\left\{b_{k}\right\}}_{k=1}^{K}$ (where is K the total number of predicted boxes in a single batch) and a class probability vector corresponding to each bounding box $P_{k}=[p_{k,1},p_{k,2},\ldots ,p_{k,C}]$ (where C is the total number of classes in the visual diagnostic task) for the target domain image stream at the time step. For a specific class, the model c iteratively maintains the sliding statistic of $\mu_{t}^{c}$ the current step based on the global mean $\mu_{t-1}^{c}$ and standard deviation of the previous $\sigma_{t-1}^{c}$ time step: $\;\sigma_{t}^{c}$

$$\begin{array}{c} \left\{ \begin{array}{rr} \mu_{t}^{c} & =\alpha \mu_{t-1}^{c}+(1-\alpha )\frac{1}{K}\sum_{k=1}^{K}p_{k,c}\\ \sigma_{t}^{c} & =\alpha \sigma_{t-1}^{c}+(1-\alpha )\sqrt{\frac{1}{K}\sum_{k=1}^{K}{\left(p_{k,c}-\mu_{t}^{c}\right)}^{2}} \end{array}\right. \end{array}$$ (5)

The momentum factor in Equation 5 α is set to 0.99. In visual perception tasks, there is often an imbalanced distribution of instance classes within a single training batch (e.g., collective absence of a specific class in a local temporal image). An exponential moving average decay rate close to 1 can resist local data mutations at the batch level, forcing the statistic $\mu_{t}^{c}$ to $\sigma_{t}^{c}$ stably track the global long-term probability distribution of the target dataset, thereby ensuring the smoothness and robustness of the dynamic threshold evolution. Based on this, the c dynamic hard pseudo-label threshold of the class $\tau_{t}^{c}$ is adaptively adjusted according to the network’s current recognition ability for that class:

$$\begin{array}{c} \tau_{t}^{c}=\text{Clip}(\mu_{t}^{c}+\beta \cdot \sigma_{t}^{c},\tau_{\min},\tau_{\max}) \end{array}$$ (6)

In Equation 6, the function Clip(·) constrains the threshold $\left[\tau_{\min},\tau_{\max}\right]$ within a reasonable range $\tau_{\min}=0.5$ . The maximum *a posteriori* probability principle is set according to Bayesian Decision Theory. In multi-class classification or binary logic, when the confidence of the highest class is below 0.5, the feature loses its majority dominance in the probability space. Forcing such non-dominant probabilities into hard pseudo-labels violates the mathematical and physical meaning of the decision boundary, inducing erroneous gradient back propagation $\tau_{\max}=0.95$ . The introduction of the top bound aims to offset the confidence calibration error and overconfidence phenomenon that exist in the later stages of training of deep neural networks. If no mandatory upper bound is set, allowing the dynamic threshold to approach 1.0 due to model overfitting will trigger the “starvation effect” in self-supervised learning. A large number of samples that originally had correct semantic guidance will be incorrectly intercepted, leading to a sharp decrease or even stagnation in the number of pseudo-labels generated in the target domain, disrupting the continuous learning evolution cycle. The parameter, β as a scaling factor controlling the variance margin, determines the statistical penalty strength of the dynamic threshold relative to mean drift. The module is configured β=1.0 with a statistically defined upper bound for the confidence level of one standard deviation. This configuration ensures that, under any batch distribution, the network only selects predictions from the top right of the current class’s probability density function (i.e., the high-value region within the top 16% of confidence ranking). This dynamic truncation based on standard deviation maximizes the removal of ambiguous background noise while maintaining a stable and pure supply of pseudo-label signals during self-supervised evolution. The class index with the highest confidence level for the current bounding box is set. When its highest predicted probability $c^{*}=\arg\max_{c}p_{k,c}p_{k,c^{*}}>\tau_{t}^{c^{*}}$ is reached, the bounding box $b_{k}$ is converted into a hard pseudo-label and stored in the high-confidence memory.

Although dynamic thresholding reduces background noise, it is prone to misidentifying actual targets that undergo abrupt morphological changes. Among alternative semi-supervised learning solutions, soft labels or consistency regularization based on the Exponential Moving Average is commonly utilized to alleviate the confirmation bias of hard labels. These methods lack explicit topological constraints on target geometric boundaries when confronted with severe spatial deformations induced by domain shifts, often propagating incomplete features of deformed targets as valid signals during backpropagation. The module further introduces a bidirectional spatial recovery mechanism using the Intersection over Union (IoU). For the set of low-confidence predicted boxes intercepted by the dynamic threshold, $B_{\mathrm{low}}$ the spatial overlap matrix between $b_{\mathrm{low}}\in B_{\mathrm{low}}$ them and the set of high-confidence pseudo-labels is calculated. Let the $B_{\text{high}}$ corresponding $b_{\text{high}}\in B_{\text{high}}$ class probability vectors be $P_{\mathrm{low}}$ and, respectively $P_{\text{high}}$ . The recovery condition requires not only spatial overlap but also approximation of the feature semantic distribution. The Jensen-Shannon divergence is used to measure the difference in the logistic distribution of the two predicted box categories, specifically defined as follows:

$$\begin{array}{c} \mathrm{JSD}(P_{\mathrm{low}}\|P_{\text{high}})=\frac{1}{2}D_{\mathrm{KL}}(P_{\mathrm{low}}\|M)+\frac{1}{2}D_{\mathrm{KL}}(P_{\text{high}}\|M) \end{array}$$ (7)

In Equation 7 $M=\frac{1}{2}(P_{\mathrm{low}}+P_{\text{high}})$ , $D_{\mathrm{KL}}$ is the relative entropy. If there exists a condition $b_{\mathrm{low}}\in B_{\mathrm{low}}$ that satisfies $\max_{b_{\text{high}}}\mathrm{IoU}(b_{\mathrm{low}},b_{\text{high}})\in [T_{\mathrm{iou}}^{\mathrm{low}},T_{\mathrm{iou}}^{\text{high}}]$ and $\mathrm{JSD}(P_{\mathrm{low}}\|P_{\text{high}})<\tau_{\mathrm{js}}$ , then the $b_{\mathrm{low}}$ category of is forcibly aligned to $b_{\text{high}}$ and restored to the supervision signal set $\left[T_{\mathrm{iou}}^{\mathrm{low}},T_{\mathrm{iou}}^{\text{high}}\right]$ . The setting of the interval follows the spatial feature matching logic and morphological distortion tolerance in target detection, and the module is strictly configured as follows [0.4,0.7] . The lower bound is set to 0.4, slightly lower than the boundary of the normal positive sample allocation (usually 0.5), which aims to give the model the necessary spatial displacement tolerance. Under extreme weather conditions, the visual boundary of the target often shifts locally due to halo, rain and fog. The bottom line of 0.4 can effectively capture samples with slight morphological distortion and isolate background noise that is disjoint from the target. The upper bound is set to 0.7, the core purpose of which is to avoid the spatial aggregation redundancy brought about by the network regression mechanism. When the overlap exceeds 0.7, the low confidence prediction box is essentially just a high-density resampling of the high confidence benchmark box. Forcibly restoring it will cause the gradient update of the spatial region to generate local overload, which in turn will cause overfitting of specific features. The parameter, $\tau_{\mathrm{js}}$ serving as the maximum tolerance threshold for logical divergence, has a limit of 0.2 established on the semantic boundary defense of consistency regularization. The Jensen-Shannon divergence is symmetric and has a bounded range; the divergence upper limit of 0.2 allows for a reasonable decrease in the probability response of a target during cross-domain migration (such as a decrease in the absolute value of confidence due to environmental noise), but prohibits cross-class semantic drift in the topology of the probability distribution.

### 3.3 Domain incremental continuous learning and structured predictive distillation mechanism

When the model undergoes continuous self-supervised iterations using pseudo-labels in the target domain, the network weights tend to shift toward the local optima of the target distribution. This parameter update is often accompanied by a decline in the model’s perceptual performance within the previous source domain scenarios, manifesting as a certain degree of knowledge forgetting. In the context of this study, “continuous learning” is defined as an online incremental learning process driven by data streams from the target domain, where the model updates its parameters dynamically without relying on the long-term storage of large-scale historical source domain samples. Existing predictive distillation research for object detection indicates that, without retaining historical data, teacher-student parameter constraints help maintain the generalization boundary of the old domain (Gang et al., 2022). Distinct from standard unsupervised domain adaptation paired with anti-forgetting regularization (which frequently employs an offline process of generating pseudo-labels followed by staged fine-tuning), this framework explores a deeply coupled path of dynamic pseudo-label generation and structured predictive distillation. Online pseudo-labels participate in guiding the adaptive adjustment of the feature space, while the teacher-student architecture synchronously applies gradient constraints during the parameter update phase. This collaborative mechanism aims to enable the model to assimilate new distribution features while reducing the risk of historical perception experience degradation from a parameter optimization perspective, helping to mitigate the potential gradient conflicts that isolated modules might face when processing non-stationary data streams.

The baseline model trained $F_{\mathrm{tea}}$ in the source domain is defined as the teacher network $D_{S}$ , whose parameter dictionary $\theta_{\mathrm{old}}$ is frozen to represent historical knowledge anchors; $D_{T}$ the model performing incremental iterations on the target domain flow is defined as the student network $F_{\mathrm{stu}}$ , whose parameter dictionary $\theta_{\mathrm{new}}$ is updated in real time with the gradient. Let the target domain input image tensor be $x_{T}$ , and the total loss function of the adaptive perception system $L_{\text{total}}$ be decoupled into the detection regression loss and the knowledge distillation penalty term:

$$\begin{array}{c} L_{\text{total}}=L_{\det}(F_{\mathrm{stu}}(x_{T}),{\hat{Y}}_{\text{pseudo}})+\lambda \cdot L_{\mathrm{kd}}(F_{\mathrm{stu}}(x_{T}),F_{\mathrm{tea}}(x_{T})) \end{array}$$ (8)

In Equation 8, λ is the knowledge distillation weight coefficient, responsible for the gradient constraint between learning new features in the target domain (plasticity) and preserving historical knowledge in the source domain (stability). In this study, it is statically configured to 1.0. The mathematical basis for this setting is to ensure that the detection loss gradient generated by cross-domain feature alignment and the distillation penalty gradient aimed at suppressing forgetting are maintained at the same order of magnitude during backpropagation, so as to smoothly absorb the environmental distribution features of the new domain without causing destructive coverage of the old domain feature decision boundary. The detection loss ${ℒ}_{\det}$ uses the dynamic hard pseudo-labels generated by the preceding module ${\hat{Y}}_{\text{pseudo}}$ to guide the student network to complete the feature alignment and parameter iteration of the target domain. Let the total number of spatial grid nodes output by the feature pyramid be N , and the number of positive sample nodes successfully assigned pseudo-labels in a single batch be $N_{\mathrm{pos}}$ . i the binary prediction confidence and bounding box coordinates of the student network for the pseudo-label foreground target at the grid are denoted as $p_{i}^{\mathrm{stu}}$ and, respectively $b_{i}^{\mathrm{stu}}$ , and the corresponding pseudo-label supervision signals are denoted as ${\hat{p}}_{i}^{\text{pseudo}}$ and ${\hat{b}}_{i}^{\text{pseudo}}$ . ${ℒ}_{\det}$ the specific formal definition of is as follows:

$$\begin{array}{c} \begin{array}{l} L_{\det}=\frac{1}{N_{\mathrm{pos}}}\\ \;\;\;\;\;\;\sum_{i=1}^{N}[L_{\mathrm{BCE}}(p_{i}^{\mathrm{stu}},{\hat{p}}_{i}^{\text{pseudo}})+I_{i}^{\mathrm{obj}}\cdot \lambda_{\text{giou}}L_{\text{GIoU}}(b_{i}^{\mathrm{stu}},{\hat{b}}_{i}^{\text{pseudo}})] \end{array} \end{array}$$ (9)

In ${ℒ}_{\mathrm{BCE}}$ Equation 9, is the Binary Cross Entropy loss, used to apply global class logic constraints to all positive and negative sample nodes. The indicator function $I_{i}^{\mathrm{obj}}\in \{0,1\}$ controls the computational boundary of the regression loss: is true if and only if the spatial grid i hits a valid pseudo-label (i.e., an anchor point determined to contain the target); $I_{i}^{\mathrm{obj}}=1$ otherwise, it is set to zero to isolate coordinate perturbations from background noise. ${ℒ}_{\text{GIoU}}$ is the Generalized Intersection over Union (IoU) loss, used to measure the scale deviation and non-overlap penalty between the predicted bounding box and the pseudo-label bounding box; $\lambda_{\text{giou}}$ is the scale coefficient balancing the classification and regression gradients.

The binary cross-entropy loss ${ℒ}_{\mathrm{BCE}}$ is responsible for aligning logical responses in the probability space, and its discrete mathematical form is defined in Equation 10:

$$\begin{array}{c} \begin{array}{l} L_{\mathrm{BCE}}(p_{i}^{\mathrm{stu}},{\hat{p}}_{i}^{\text{pseudo}})=\\ -[{\hat{p}}_{i}^{\text{pseudo}}\log(p_{i}^{\mathrm{stu}})+(1-{\hat{p}}_{i}^{\text{pseudo}})\log(1-p_{i}^{\mathrm{stu}})] \end{array} \end{array}$$ (10)

The Generalized Intersection over Union loss ${ℒ}_{\text{GIoU}}$ GIoU) improves upon the gradient-vanishing issue of the traditional IoU loss when the predicted bounding box and the pseudo-label bounding box do not overlap by introducing a minimum enclosing convex-hull penalty term. Based on the aforementioned formula, $b_{i}^{\mathrm{stu}}$ the spatial intersection area of the student network’s predicted bounding box I and the pseudo-label reference box is given by, the ${\hat{b}}_{i}^{\text{pseudo}}$ union area by U , and the area of their minimum bounding rectangle in the two-dimensional coordinate system by $A^{c}$ . The formula for calculating this loss term is defined in Equation 11 as follows:

$$\begin{array}{c} L_{\text{GIoU}}(b_{i}^{\mathrm{stu}},{\hat{b}}_{i}^{\text{pseudo}})=1-(\frac{I}{U}-\frac{A^{c}-U}{A^{c}}) \end{array}$$ (11)

When the regression penalty term has an intersection, it measures the spatial overlap. At the same time, in the discrete state where they do not overlap at all, $\frac{A^{c}-U}{A^{c}}$ the term provides a smooth and continuous distance-closing gradient, which optimizes the scale morphology approximation trajectory of edge-distorted targets in complex diagnostic scenarios.

To preserve the network’s response logic to common features, the distillation loss ${ℒ}_{\mathrm{kd}}$ is subdivided into logistic classification distillation ${ℒ}_{\mathrm{cls}\_\mathrm{kd}}$ and structured regression distillation ${ℒ}_{\mathrm{reg}\_\mathrm{kd}}$ . In the classification branch, the unactivated logistic values (Logits) output by the model contain rich inter-class dark knowledge. Let i the Logits output $z_{i,c}^{\mathrm{tea}}$ by the teacher network and the student network for each class at the feature map location be c and, respectively $z_{i,c}^{\mathrm{stu}}$ . A temperature parameter is introduced T to smooth the probability distribution of all classes. Let the i-th elements of the multi-class probability distributions output by the teacher and student networks be $p_{i,c}^{\mathrm{tea}}$ and $p_{i,c}^{\mathrm{stu}}$ , respectively; these softened probability distributions are defined in Equation 12:

$$\begin{array}{c} \left\{ \begin{array}{rr} p_{i,c}^{\mathrm{tea}} & =\frac{\exp(z_{i,c}^{\mathrm{tea}}/T)}{\sum_{j=1}^{C}\exp(z_{i,j}^{\mathrm{tea}}/T)}\\ p_{i,c}^{\mathrm{stu}} & =\frac{\exp(z_{i,c}^{\mathrm{stu}}/T)}{\sum_{j=1}^{C}\exp(z_{i,j}^{\mathrm{stu}}/T)} \end{array}\right. \end{array}$$ (12)

The corresponding classification distillation loss is then calculated according to Equation 13:

$$\begin{array}{c} L_{\mathrm{cls}\_\mathrm{kd}}=\frac{1}{N\cdot C}\sum_{i=1}^{N}\sum_{c=1}^{C}(p_{i,c}^{\mathrm{tea}}\log\frac{p_{i,c}^{\mathrm{tea}}}{p_{i,c}^{\mathrm{stu}}})\cdot T^{2} \end{array}$$ (13)

In the above formula, C represents the total number of categories in the object detection task, and N represents the total number of spatial grid nodes participating in feature distillation T . The introduction of temperature effectively amplifies the implicit correlations between non-target categories, forcing the student network to inherit the deep classification topology boundaries of the old domain while approximating the features of the new domain.

In the feature regression branch, background pixels from different domains often exhibit significant semantic spans. Directly applying L1 distance constraints on bounding box regression features to all pixels on the feature map can lead to gradient conflicts between extracting new features from the target domain and preserving old knowledge. Let i the bounding box regression features of the student network and the teacher network at the spatial grid nodes be denoted as $R_{i}^{\mathrm{stu}}$ and, respectively $R_{i}^{\mathrm{tea}}$ . The model introduces a spatial attention mask matrix $M_{\text{mask}}$ and the structured regression distillation loss, as defined in Equation 14:

$$\begin{array}{c} \left\{ \begin{array}{rr} M_{\text{mask}}(i) & =I(\max_{c}p_{i,c}^{\mathrm{tea}}>\tau_{\text{mask}})\\ L_{\mathrm{reg}\_\mathrm{kd}} & =\frac{\sum_{i=1}^{N}M_{\text{mask}}(i)\cdot R_{i}^{\mathrm{stu}}-{R_{i}^{\mathrm{tea}}}_{1}}{\sum_{i=1}^{N}M_{\text{mask}}(i)+\in_{2}} \end{array}\right. \end{array}$$ (14)

The indicator function I(·) forces the mask values of worthless background regions to zero based on the confidence output of $\tau_{\text{mask}}$ the teacher network at spatial coordinates. The parameter i is set to a low-sensitivity threshold of 0.1. This value is not used in the traditional high-confidence truncation of 0.5 because target edges and difficult negative sample regions (i.e., the transition region with confidence between 0.1 and 0.5) contain crucial morphological evolution information. This masking mechanism filters out interference from pure environmental backgrounds (confidence approaching zero) that do not contribute to the diagnostic task, while focusing regression distillation on anchor regions with entity information and geometric features. A small constant introduced in the denominator $\epsilon_{2}$ (with a value of $1\times 10^{-6}$ ) ensures the numerical stability of the L1 norm operation when the mask tends to be empty.

### 3.4 Overall optimization framework and algorithm implementation

The entire complex scene adaptive network operates in an end-to-end online learning mode. Forward propagation of the target domain image stream simultaneously drives pseudo-label generation and knowledge distillation error feedback. The overall parameter optimization trajectory of the algorithm relies on gradient descent. After the student network completes the forward feature mapping of the current batch, its parameters are updated using the AdamW optimizer according to Equation 15:

$$\begin{array}{c} \theta_{\mathrm{new}}\leftarrow \theta_{\mathrm{new}}-\eta_{\mathrm{lr}}\nabla_{\theta_{\mathrm{new}}}L_{\text{total}} \end{array}$$ (15)

Here $\eta_{\mathrm{lr}}$ , represents the adaptively adjusted learning rate. To ensure the smooth evolution of the knowledge system and prevent the student network from being damaged by instantaneous gradients from extremely noisy images, the teacher network does not participate in backpropagation but instead relies on the Exponential Moving Average of the student network for soft parameter updates. Let the momentum decay coefficient be $\eta_{\mathrm{ema}}$ . At the end of each iteration, the teacher-network parameters $\theta_{\mathrm{old}}$ are updated according to Equation 16:

$$\begin{array}{c} \theta_{\mathrm{old}}\leftarrow \eta_{\mathrm{ema}}\theta_{\mathrm{old}}+(1-\eta_{\mathrm{ema}})\theta_{\mathrm{new}} \end{array}$$ (16)

High-confidence $\eta_{\mathrm{ema}}$ momentum decay (typically close to 0.999) facilitates the teacher model in smoothly absorbing the feature representations of new domains. Pseudo-label generation, predicted feature extraction, and distillation matching share the forward computation flow of the backbone network, avoiding exponential memory and computational consumption during the forward propagation phase of the training cycle. This coupled architecture mitigates the conflict between extracting new features and forgetting old experiences in continuous visual diagnosis, constructing a long-cycle, high-precision adaptive perception evolution framework on edge computing nodes with limited hardware resources.

## 4 Mathematical definition of dataset analysis and evaluation indicators

### 4.1 Adaptive datasets for complex scenes

The ability of visual diagnostic models to generalize to heterogeneous environments relies on data distributions that include real-world physical distortions. To verify the feature repair capabilities of visual diagnostic systems in dealing with real-world physical domain offsets, this study selects highly dynamic autonomous driving scenarios with environmental distortions as the test subject. The BDD100K large-scale panoramic driving dataset is used as the core experimental benchmark. This dataset covers complex urban dynamic scenes and provides accurate metadata labels (such as weather conditions, ambient lighting, and time periods). In terms of category distribution, this study focuses on core interactive entities in dynamic scenes, covering major categories such as cars, pedestrians, buses, trucks, motorcycles, bicycles, traffic lights, and traffic signs, providing a physical foundation for constructing cross-domain adaptive tasks.

To verify the domain shift effect of illumination and weather conditions, the experiment divided the data into independent source and target domains based on attribute labels. In the illumination transfer task, 36,728 samples from daytime images were extracted to construct the source domain for fully supervised prior feature extraction of the model; 27,971 samples from nighttime images were extracted, and after removing labels, the target domain was constructed for unsupervised adaptive evaluation, with 3,929 labeled nighttime images retained as an independent test set. In the weather transfer task, 42,351 samples from clear weather data were used as the source domain, and 10,420 samples from low visibility data such as rain/foggy were used as the target domain for unsupervised evolution, with 1,460 rain/foggy images used for final performance evaluation. This structured domain decoupling strategy with a clearly defined data scale isolates the distribution homology between training and testing, thus objectively mapping the adaptive perception boundary of the visual diagnostic system in a real open environment. This study additionally introduces the Cityscapes to Foggy Cityscapes meteorological transfer benchmark. This benchmark utilizes 2,975 urban scene images from Cityscapes as the source domain, and the corresponding Foggy Cityscapes synthetic fog images as the target domain, to test the model’s generalization across different physical acquisition devices.

### 4.2 Rigorous mathematical definition of evaluation indicators

To quantify the comprehensive performance of the adaptive diagnostic model in multi-scale feature parsing and cross-domain classification, the following evaluation metrics were adopted. The perceptual performance of the model is evaluated using the mean Average Precision at a single IoU threshold (mAP@0.5) and the mean Average Precision averaged across multiple IoU thresholds from 0.5 to 0.95 with a step size of 0.05 (mAP@0.5:0.95). Integrating mAP@0.5:0.95 provides a multi-dimensional reference for assessing the bounding box regression quality. The underlying calculation of this metric is based on the fundamental mathematical mapping of Intersection over Union (IoU), Precision, P and Recall R .

Intersection over Union (IoU) measures the geometric overlap between the predicted bounding box $B_{p}$ and the ground-truth bounding box $B_{\mathrm{gt}}$ as defined in Equation 17:

$$\begin{array}{c} \mathrm{IoU}=\frac{\text{Area}(B_{p}\cap B_{\mathrm{gt}})}{\text{Area}(B_{p}\cup B_{\mathrm{gt}})} \end{array}$$ (17)

Given the IoU threshold constraint, the probabilistic definitions of precision Prec and recall Rec are given in Equation 18:

$$\begin{array}{c} \left\{ \begin{array}{c} \text{Prec}=\frac{\mathrm{TP}}{\mathrm{TP}+\mathrm{FP}}\\ \mathrm{Rec}=\frac{\mathrm{TP}}{\mathrm{TP}+\mathrm{FN}} \end{array}\right. \end{array}$$ (18)

For a specific diagnostic target category c , the average precision $AP_{c}$ is defined as the area under the precision-recall curve, as given in Equation 19:

$$\begin{array}{c} AP_{c}=\int_{0}^{1}P_{c}(R_{c})dR_{c} \end{array}$$ (19)

The system’s global perception capability over all C diagnostic categories is represented by mAP, as defined in Equation 20:

$$\begin{array}{c} \mathrm{mAP}=\frac{1}{C}\sum_{c=1}^{C}AP_{c} \end{array}$$ (20)

Furthermore, in self-supervised mechanisms involving dynamic threshold truncation and pseudo-label filtering, a “starvation phenomenon” often occurs, where the model achieves high precision but suffers a significant drop in recall. To evaluate the balance between precision and recall, the F1−Score is introduced as their harmonic mean, as defined in Equation 21:

$$\begin{array}{c} F1-\text{Score}=2\cdot \frac{\text{Prec}\cdot \mathrm{Rec}}{\text{Prec}+\mathrm{Rec}} \end{array}$$ (21)

This indicator suppresses statistical bias caused by single-dimensional extrema, providing a comprehensive assessment of the model’s perceptual performance.

In addition to spatial awareness accuracy, the computational complexity assessment for edge deployment introduces global floating-point operation time (FLOPs) and model parameter count (Params) as static quantitative benchmarks for network topology capacity. For dynamic inference efficiency metrics, the calibration of single-image processing latency and frame rate throughput (FPS) is decoupled from the computational power of the graphics processor cluster and constrained to execution on general-purpose x86 physical computing nodes running the Ubuntu operating system and not relying on dedicated tensor acceleration instruction sets. This ensures that experimental data accurately reflects the model’s engineering applicability on resource-constrained edge devices.

### 4.3 Experimental configuration

The baseline model in this study is designated as YOLOv5s. The network architecture and fundamental training pipeline are constructed based on the official Ultralytics YOLOv5 v7.0 repository. During the initialization phase, the model utilizes pre-trained weights from the MS COCO dataset to provide a stable initial feature mapping space.

The development and evaluation of the algorithm are deployed on the Ubuntu operating system. During the parameter optimization phase, the model training is accelerated by an NVIDIA GeForce RTX 3060 GPU with 12 GB of graphics memory, utilizing the PyTorch 1.13.1 deep learning framework and CUDA version 11.7. To evaluate the model’s engineering applicability on resource-constrained edge devices, the inference framework is decentralized to a general-purpose physical computing node. This underlying evaluation node is equipped with an Intel Core i5 processor and 16 GB of memory, relying on Python 3.8 and OpenCV to execute cross-domain data stream processing and perception inference. The network employs the AdamW optimizer with a designated weight decay coefficient of 0.0005.

Regarding the training cycle planning, the model undergoes 100 pre-training epochs on the source domain data. In the subsequent target domain adaptation phase, the continuous learning process executes 10 incremental iterations per round. To maintain the stability of historical knowledge and ensure a smooth parameter evolution, the weight update of the teacher network adopts the Exponential Moving Average mechanism, with a momentum coefficient configured at 0.999.

## 5 Experimental results and analysis

### 5.1 Cross-domain target detection comparative experiment

**Figure 3:**
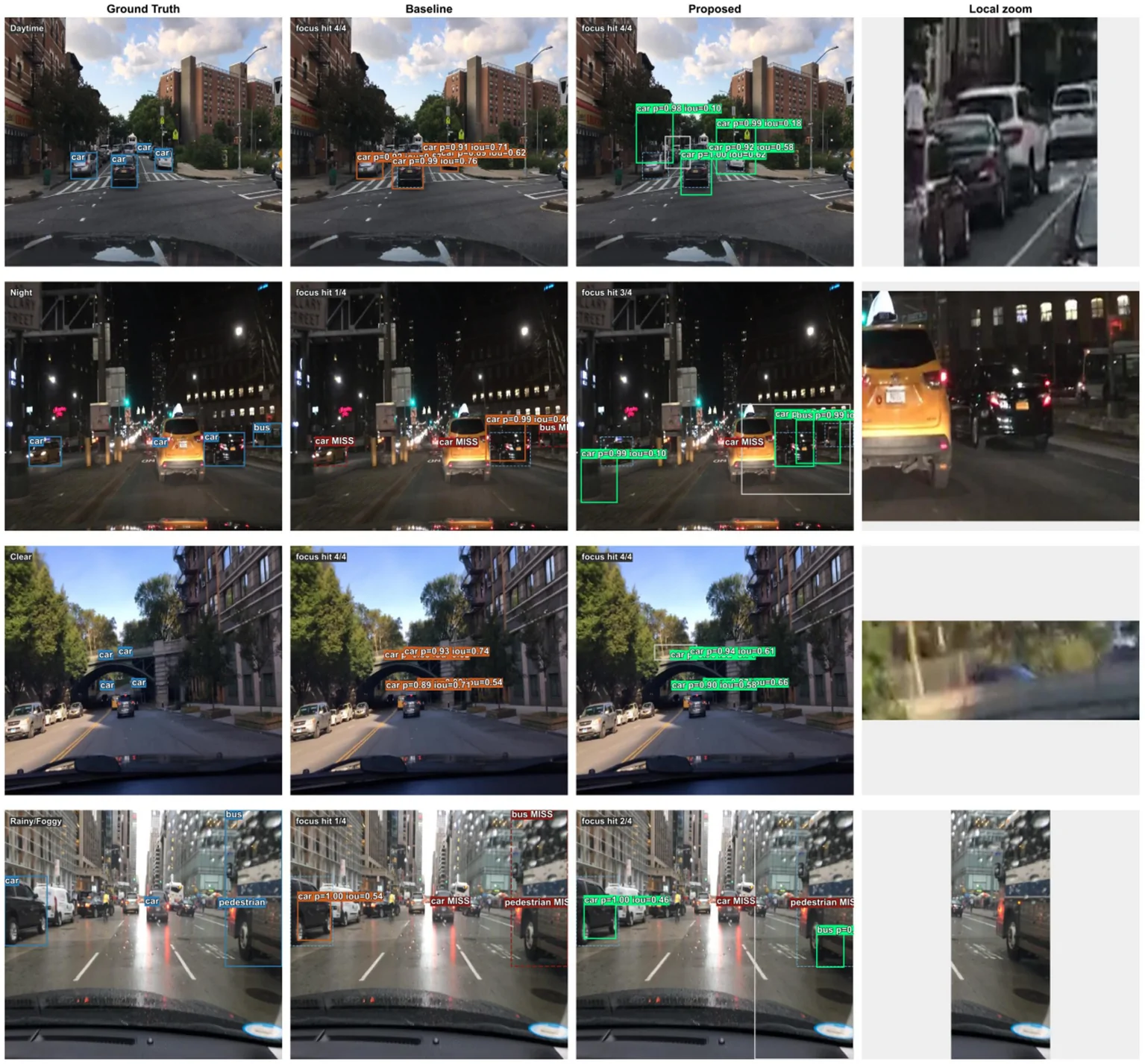
Comparison of visual detection performance under complex-environment transfer tasks. The first two rows correspond to daytime-to-nighttime illumination transfer, and the last two rows correspond to clear-to-rainy/foggy weather transfer; columns show Ground Truth, Baseline, Proposed, and Local zoom. Figure adapted from Yu, et al. (2020). https://bdd-data.berkeley.edu/download.html.

To evaluate the cross-domain perception performance of the model in open environments with light variations and weather changes, this section outlines target detection experiments based on physical environment shifts. The experiment utilizes the BDD100K dataset and sets up two domain transfer tasks: light shift (daytime to nighttime) and weather shift (clear to rain/fog). The source domain contains pixel-level annotations to initialize the feature mapping space; the target domain uses an unlabeled image stream to observe unsupervised environmental adaptability. The experiment includes tests on BDD100K and the Cityscapes to Foggy Cityscapes benchmark to cover different distributions. The evaluation selects standard YOLOv5 as the baseline detector and includes recent state-of-the-art unsupervised domain adaptive methods published in the past 3 years (such as SSDA-YOLO, DA-YOLOv8, and D-Know) for comparison. To quantify the perception performance and computational efficiency, the evaluation employs mean average precision (mAP@0.5, mAP@0.5:0.95), the harmonic index (F1-Score), floating-point operations (FLOPs), and inference frame rate (FPS) as metrics. The quantitative statistics and visual comparison results are presented in Table 1 and Figure 3.

**Table 1:** Comparison of perception performance and computational efficiency among different algorithms across domain migration tasks on BDD100K and Cityscapes datasets.

Method	Source domain	Target domain	Physical environment shift	mAP@0.5 (%)	mAP@0.5:0.95 (%)	F1-Score	FLOPs (G)	FPS
Standard YOLOv5 Baseline	Daytime Panoramic (Daytime)	Low-light Nighttime (Night)	Severe Photometric Shift and Artificial Light Interference	31.2	14.5	0.335	15.8	20.6
SSDA-YOLO	Daytime Panoramic (Daytime)	Low-light Nighttime (Night)	Severe Photometric Shift and Artificial Light Interference	38.1	18.5	0.402	16.2	19.8
DA-YOLOv8	Daytime Panoramic (Daytime)	Low-light Nighttime (Night)	Severe Photometric Shift and Artificial Light Interference	39.5	19.4	0.415	16.3	19.7
D-Know	Daytime Panoramic (Daytime)	Low-light Nighttime (Night)	Severe Photometric Shift and Artificial Light Interference	40.7	20.3	0.428	16.5	19.5
Proposed Adaptive Dual-Net	Daytime Panoramic (Daytime)	Low-light Nighttime (Night)	Severe Photometric Shift and Artificial Light Interference	43.5	22.1	0.465	16.6	19.3
Standard YOLOv5 Baseline	High-Visibility Clear (Clear)	Severe Rain/Fog Occlusion (Rainy/Foggy)	High-Frequency Weather Scattering and Target Distortion	34.6	16.8	0.375	15.8	20.6
SSDA-YOLO	High-Visibility Clear (Clear)	Severe Rain/Fog Occlusion (Rainy/Foggy)	High-Frequency Weather Scattering and Target Distortion	41.5	20.7	0.430	16.2	19.8
DA-YOLOv8	High-Visibility Clear (Clear)	Severe Rain/Fog Occlusion (Rainy/Foggy)	High-Frequency Weather Scattering and Target Distortion	43.0	21.6	0.445	16.3	19.7
D-Know	High-Visibility Clear (Clear)	Severe Rain/Fog Occlusion (Rainy/Foggy)	High-Frequency Weather Scattering and Target Distortion	44.2	22.4	0.461	16.5	19.5
Proposed Adaptive Dual-Net	High-Visibility Clear (Clear)	Severe Rain/Fog Occlusion (Rainy/Foggy)	High-Frequency Weather Scattering and Target Distortion	47.1	24.5	0.492	16.6	19.3
Standard YOLOv5 Baseline	Clear Urban (Cityscapes)	Dense Fog (Foggy Cityscapes)	Synthetic Weather Scattering	35.5	17.2	0.382	15.8	20.6
SSDA-YOLO	Clear Urban (Cityscapes)	Dense Fog (Foggy Cityscapes)	Synthetic Weather Scattering	42.1	21.0	0.436	16.2	19.8
DA-YOLOv8	Clear Urban (Cityscapes)	Dense Fog (Foggy Cityscapes)	Synthetic Weather Scattering	43.8	22.0	0.450	16.3	19.7
D-Know	Clear Urban (Cityscapes)	Dense Fog (Foggy Cityscapes)	Synthetic Weather Scattering	45.4	22.9	0.472	16.5	19.5
Proposed Adaptive Dual-Net	Clear Urban (Cityscapes)	Dense Fog (Foggy Cityscapes)	Synthetic Weather Scattering	48.3	25.1	0.505	16.6	19.3

Quantitative evaluation reveals the differences in characteristic representations of different network topologies when coping with abrupt changes in physical rendering conditions. As shown in Table 1, under data distribution shifts, the basic YOLOv5 model experiences degradation in generalization ability. Its mAP@0.5 and the strict mAP@0.5:0.95 drop to 31.2% and 14.5% in the nighttime domain, and 34.6% and 16.8% in the rain/fog target domain, respectively. The comparative methods (SSDA-YOLO, DA-YOLOv8, and D-Know) mitigate the domain shift effect through feature alignment or adversarial learning. The D-Know algorithm achieves an mAP@0.5:0.95 of 20.3% and 22.4% in the illumination and weather migration tasks. The proposed adaptive dual network records detection accuracies (mAP@0.5:0.95) of 22.1% and 24.5% in these two tasks. In the cross-dataset transfer test from Cityscapes to Foggy Cityscapes, the proposed model reaches an mAP@0.5:0.95 of 25.1%. All evaluated models maintain comparable inference efficiency, with FLOPs distributed in the range of 15.8–16.6 GFLOPs and FPS remaining above 19.3, satisfying the hardware constraints for edge deployment. The adaptive dual-network model constructed in this study, without accessing target domain labels, records an mAP@0.5 of 48.3% and an F1-Score of 0.505. This represents an improvement of 2.9% over the D-Know algorithm and 12.8% over the baseline model. Data testing independent of BDD100K proves that the spatial recovery mechanism and distillation constraints embedded in the framework maintain an objective feature compensation utility when processing heterogeneous physical noise distributions.

Analysis of the two-dimensional spatial mapping results in Figure 3 shows that the baseline model suffers from relatively severe semantic feature attenuation in nighttime strong backlight areas and high-frequency scattering scenarios such as rain and fog, leading to large-scale missed detections of distant small entities, pedestrian groups, and overlapping targets (marked with red MISS in the figure). The proposed system, leveraging the cross-scale feature compensation and low-confidence bidirectional spatial recovery mechanism of the BiFPN architecture, captures complex targets exhibiting local morphological distortions caused by water mist halos and non-uniform light sources. Its output predicted bounding boxes not only achieve spatial fit (marked with green high-confidence in the figure) but also filter out high-frequency artifact noise from road surface diffuse reflection without introducing any manual supervision signals from the target domain. The cross-verification of the above quantitative indicators and visual representations demonstrates that the system design of this adaptive framework at the pixel-level feature fusion and gradient alignment levels improves the upper limit of high signal-to-noise ratio extraction for visual perception models in non-stationary domains.

### 5.2 Domain incremental continuous learning anti-forgetting experiment

Visual perception systems in open environments often face the degradation of historical scene feature representations when adapting to new data distributions. To verify the effectiveness of the domain incremental continuous learning mechanism based on predictive distillation in suppressing knowledge forgetting, this section constructs a sequential domain incremental learning validation. The experiment sets the base model to undergo supervised pre-training in the source domain, followed by multiple rounds of adaptive iteration on a continuously input unlabeled data stream in the target domain. At the end of each incremental weight update cycle, the current network parameters are frozen, and feature resampling and inference are performed on a static source domain test set. The decay trajectory of the mean average precision (mAP@0.5) of the source domain is quantified and recorded. In parallel, the target-domain adaptation evolution curves for different transfer tasks are plotted to quantitatively evaluate the balance between stability and plasticity. The experiment uses a pure self-training architecture without distillation penalty as the baseline control, and introduces classic methods in the field of continuous learning—EWC (Elastic Weight Consolidation) based on parameter regularization and LwF (Learning without Forgetting) based on response distillation—for multi-track curve comparison to intuitively map the implicit knowledge consolidation capabilities of different optimization paths during continuous evolution.

**Figure 4:**
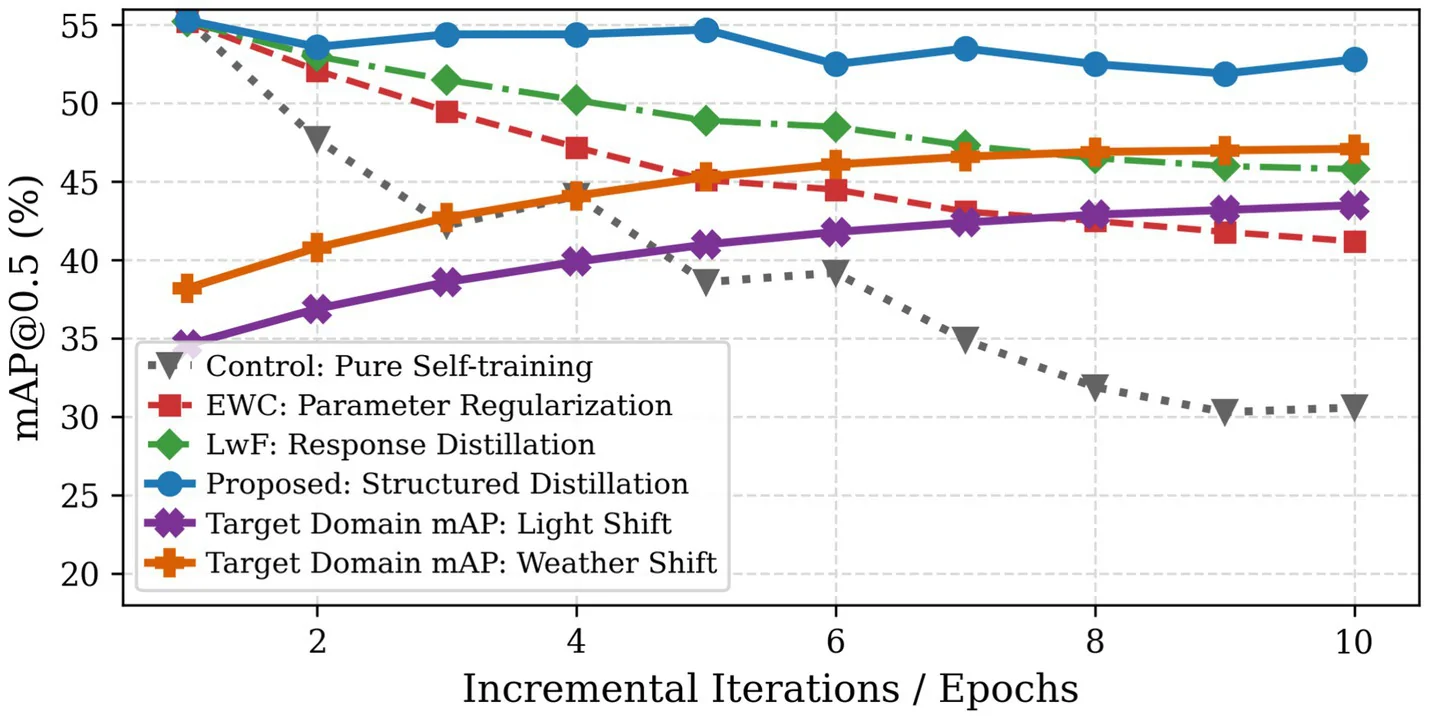
Comparison of source-domain knowledge retention (mAP@0.5) and target-domain adaptation evolution (mAP@0.5) trajectories for multiple algorithms during incremental iterations.

The evolutionary topology of the decay curves intuitively reflects the generalization maintenance boundary of different parameter update strategies in a non-stationary feature space. Observing the evolution trajectories of multiple curves in Figure 4, it can be seen that when the pure self-trained control model absorbs new distribution features in the target domain, its underlying source domain mapping weights slide towards a local suboptimal solution, causing the source domain detection accuracy to show a downward trend in the initial few learning cycles, eventually producing disordered oscillations in the low range of around 30%. The EWC method mitigates weight drift by applying a secondary penalty to key parameters. When dealing with multi-scale spatial deformation and physical photometric shift in the target detection task, due to the lack of adaptive alignment at the feature level, its source domain accuracy declines in the incremental stage, settling at around 41%. The LwF algorithm relies on the cross-entropy of the output logic response to maintain historical experience, mitigating the collapse of the classification boundary. Driven by long-term unsupervised data streams, its anti-forgetting performance is constrained by the gradual accumulation of calibration errors inherent in the soft labels themselves, maintaining a level of around 46%.

The proposed model, incorporating a structured predictive distillation mechanism, exhibits resilience against degradation after 10 incremental adaptive cycles, maintaining an accuracy quadrant above 52% throughout. The additionally plotted target-domain mAP evolution curves map the network’s plasticity. During the continuous absorption of unlabeled data streams, the target-domain detection accuracy for the Light Shift task ascends from the initial phase of approximately 34.6%, gradually converging to 43.5%. The Weather Shift task follows a similar upward trajectory, starting from around 38.2% and reaching 47.1% by the 10th epoch. The parallel observation of the stable source-domain curve and the ascending target-domain curves indicates that the introduced distillation penalty term does not restrict the assimilation of new environmental knowledge. Introducing temperature-parameter-based classification distillation helps maintain the transfer of dark knowledge between classes; simultaneously, regression distillation guided by spatial attention masks enables the network to focus on domain-invariant geometric anchors, reducing the interference of background noise in the target domain on the feature boundaries of the old domain. This feature-preserving topology endows the model with adaptive capabilities in the new domain environment, alleviating the degradation of historical perception patterns from a parameter optimization perspective, and establishing a mathematical foundation for continuous diagnosis of edge computing nodes.

### 5.3 Ablation experiment of core components

**Figure 5:**
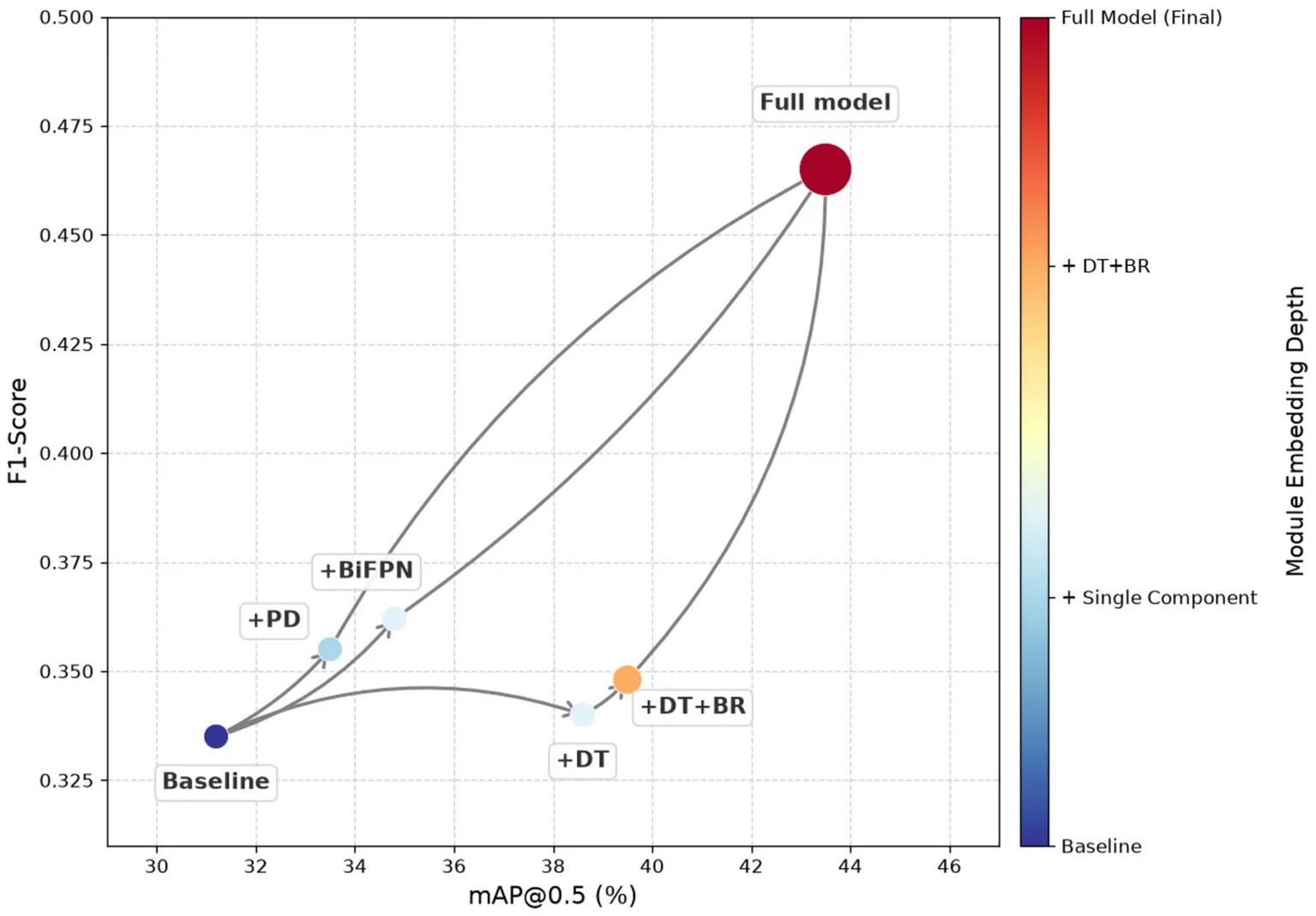
Ablation results for different module configurations. Each point represents the mAP@0.5 and F1-score of one configuration (The corresponding mAP@0.5:0.95, pseudo-label quantity, purity, FLOPs, and FPS are reported in Table 2).

To systematically evaluate the independent contributions and interactive effects of the core components within the proposed framework, this section conducts ablation experiments under a hybrid domain migration scenario. The experimental design progresses from a baseline single-stage detector to independent and combined configurations of the BiFPN multi-scale fusion module, dynamic thresholding (DT), IoU/JSD-based bidirectional recovery (BR), and predictive distillation (PD). The evaluation metrics include mean average precision (mAP@0.5, mAP@0.5:0.95), the harmonic index (F1-Score), high-confidence pseudo-label quantity, category purity, computational operations (FLOPs), and processing frame rate (FPS). The quantitative results are summarized in Table 2, and the corresponding performance distribution is illustrated in Figure 5.

**Table 2:** Ablation analysis of the independent embedding and combined configuration of core components on system perception accuracy and pseudo-label quality.

Model variant (module combination)	mAP@0.5 (%)	mAP@0.5:0.95 (%)	F1-Score	Pseudo-Label (K/Epoch)	Purity (%)	FLOPs (G)	FPS
Baseline (Base YOLO)	31.2	14.5	0.335	-	-	15.8	20.6
Baseline + BiFPN	34.8	16.7	0.362	-	-	16.6	19.8
Baseline + DT	38.6	18.2	0.340	38.4	90.1	15.8	20.2
Baseline + DT + BR	39.5	19.6	0.348	42.5	88.6	15.8	20.0
Baseline + PD	33.5	15.8	0.355	-	-	15.8	19.6
Full Model (BiFPN + DT + BR + PD)	43.5	22.1	0.465	65.8	92.4	16.6	19.3

Table 2 and Figure 5 summarize the contribution of each component under the evaluated domain-transfer setting. Adding BiFPN increases mAP@0.5 from 31.2% to 34.8%. The DT configuration reaches an mAP@0.5 of 38.6% and a pseudo-label purity of 90.1%. When BR is combined with DT, the pseudo-label quantity increases from 38.4 to 42.5 K/epoch and the F1-score increases from 0.340 to 0.348, while the purity decreases slightly to 88.6%. This result suggests a trade-off between pseudo-label coverage and purity. The PD-only configuration obtains an mAP@0.5 of 33.5% and an F1-score of 0.355. The full configuration reaches 43.5% mAP@0.5, 22.1% mAP@0.5:0.95, and an F1-score of 0.465, with a computational cost of 16.6 GFLOPs and an inference rate of 19.3 FPS. These observations indicate that the four components provide complementary contributions under the current experimental conditions.

### 5.4 Sensitivity analysis of key hyperparameters

**Figure 6:**
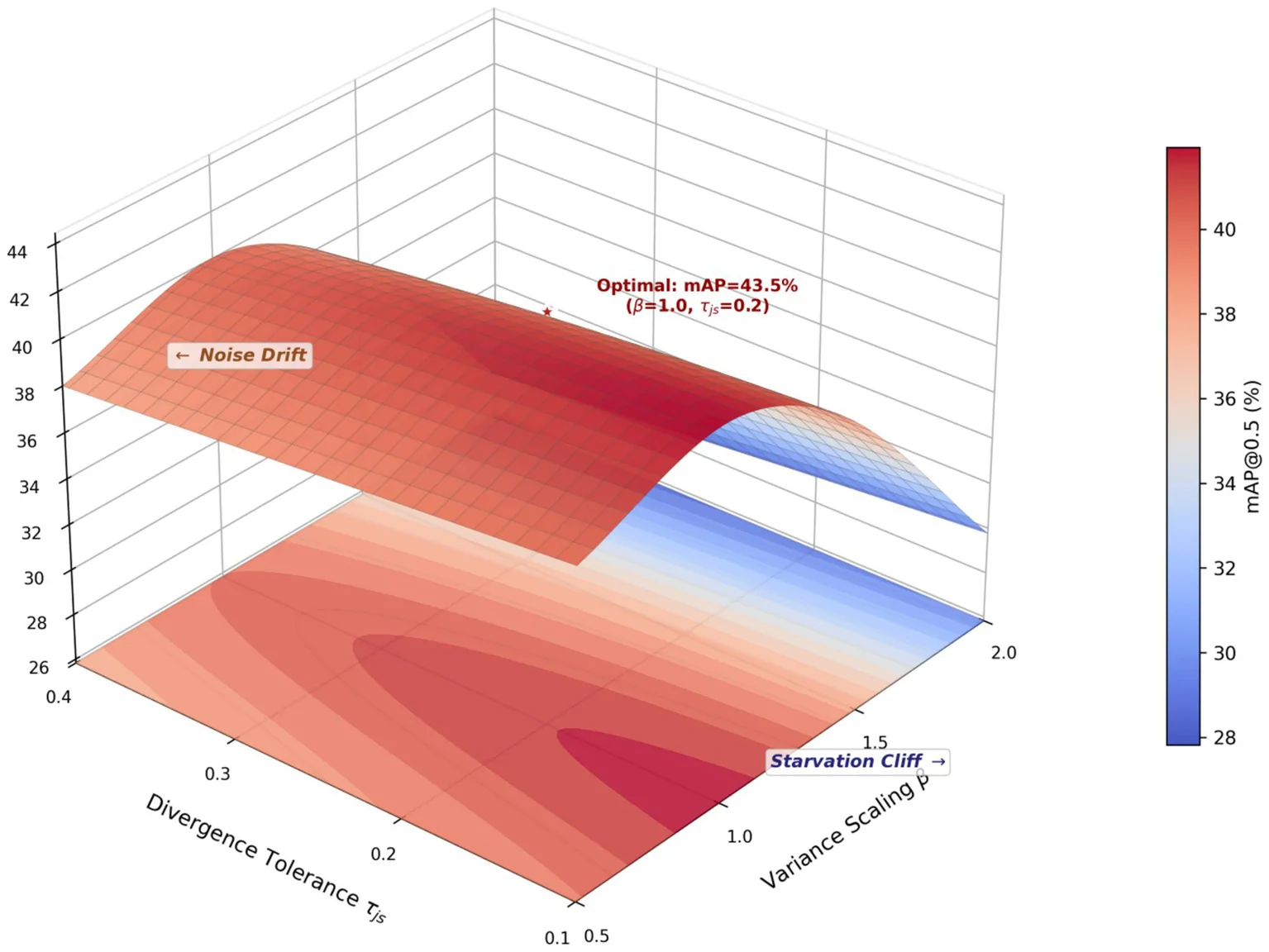
Three-dimensional sensitivity surface of mAP@0.5 with respect to the variance-scaling factor β and divergence-tolerance threshold $\tau_{\mathrm{js}}$ .

To isolate the model’s perceptual performance from the random dependence of specific empirical hyperparameters and to explore the statistical evolution of the self-supervised pseudo-label mechanism in dynamic data streams, this section conducts a two-dimensional sensitivity analysis on the core control variables of the threshold generation module. The adaptive robustness under non-stationary visual streams is highly constrained by the joint spatial game between the variance scaling factor β and the logistic divergence tolerance threshold $\tau_{\mathrm{js}}$ . The experiment relies on a drastic domain migration task with alternating day and night, employing a grid search strategy to traverse and sample the joint parameter space. The set value boundaries are [0.5,2.0] and the candidate domains are [0.1,0.4] . The evaluation system not only records the final mean accuracy (mAP@0.5) under each discrete combination configuration but also focuses on the dynamic balance between the supply rate of high-confidence pseudo-labels and class purity in the later stages of model evolution. The bivariate performance response surface after continuous interpolation fitting is shown in Figure 6.

The three-dimensional sensitivity response surface intuitively reveals the statistical constraint boundary and optimal generalization stationary point under the dual-parameter coupling effect. β observing along the variance scaling factor axis, when β approaching 2.0, the lower bound of the dynamically generated threshold is excessively raised, and the model falls into a severe “starvation cliff,” resulting in a precipitous drop in detection accuracy (below 32%) due to insufficient positive gradient excitation. Conversely, if β it drops to 0.5, the relaxed threshold boundary induces uncontrollable “noise drift,” with a large number of artifacts being mistaken for entities, causing β a significant drop in the surface in the low region. Simultaneously examining $\tau_{\mathrm{js}}$ the response gradient of the logistic divergence tolerance threshold, its physical meaning characterizes the semantic consistency defense against cross-domain feature distortion: $\tau_{\mathrm{js}}$ excessive relaxation to 0.4 weakens the high-dimensional manifold boundary between categories, inducing the mixing of outlier samples; while excessive tightening to 0.1 excludes difficult entity targets that undergo reasonable deformation. As can be seen from the convergence center of the two-dimensional contour projection, when the parameter combination is anchored at $\left(\beta =1.0,\tau_{\mathrm{js}}=0.2\right)$ the local optimal stationary point, the surface climbs to the global peak (mAP = 43.5%). This surface topology indicates that the dynamic threshold mechanism maintains robustness across different parameter combinations, balancing domain-shift denoising and feature preservation.

### 5.5 Feature space distribution and edge-side computational complexity assessment

**Figure 7:**
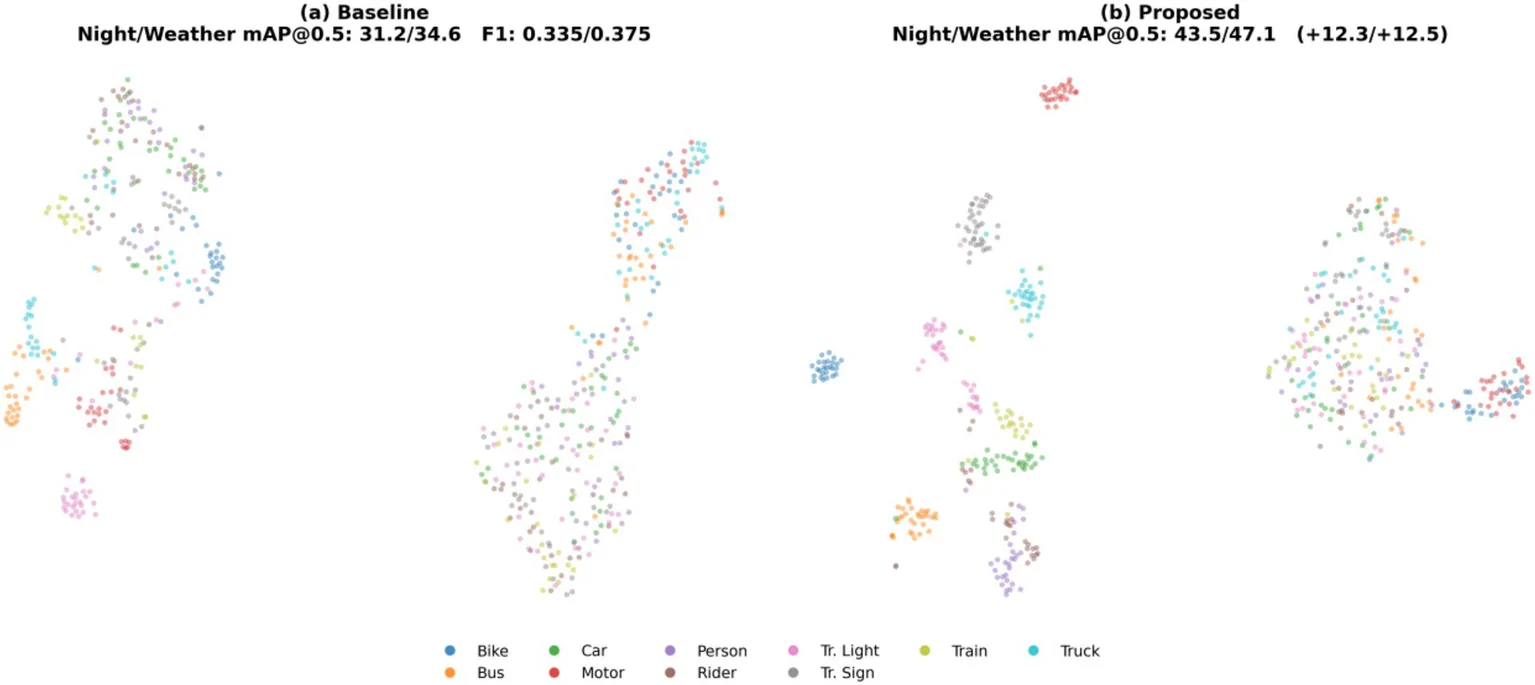
Visualization of t-SNE feature-space clustering distributions under nighttime and adverse-weather domain shifts: **(a)** baseline model; **(b)** proposed adaptive model.

The efficiency of cross-domain alignment of multi-scale features and the computational cost of lightweight architecture are key dimensions for evaluating the general applicability of diagnostic systems engineering. To intuitively verify the model’s feature decoupling capability under domain transition conditions, this section introduces t-SNE (t-Distributed Stochastic Neighbor Embedding), a dimensionality reduction technique with strong nonlinear manifold mapping properties. This algorithm can project structured information in the high-dimensional feature space onto a low-dimensional manifold, revealing the aggregation degree of feature clusters and the inter-class separation degree while preserving the local topological structure by calculating the probability distribution similarity between sample points. Using this technique to perform two-dimensional spatial mapping projection on the high-dimensional tensor of the base network and the proposed model at the BiFPN output, the clustering evolution law of features under complex domain offset environments can be clearly observed. Meanwhile, to verify the engineering assumption of “edge device deployment and long-cycle adaptive perception” mentioned earlier, this section departs from the computationally powerful GPU cluster and decentralizes the inference framework to a general-purpose computing node based on an Intel Core i5 processor and 16 GB of memory for single-threaded constraint testing. The relevant latent space distribution features are depicted in Figure 7.

**Figure 8:**
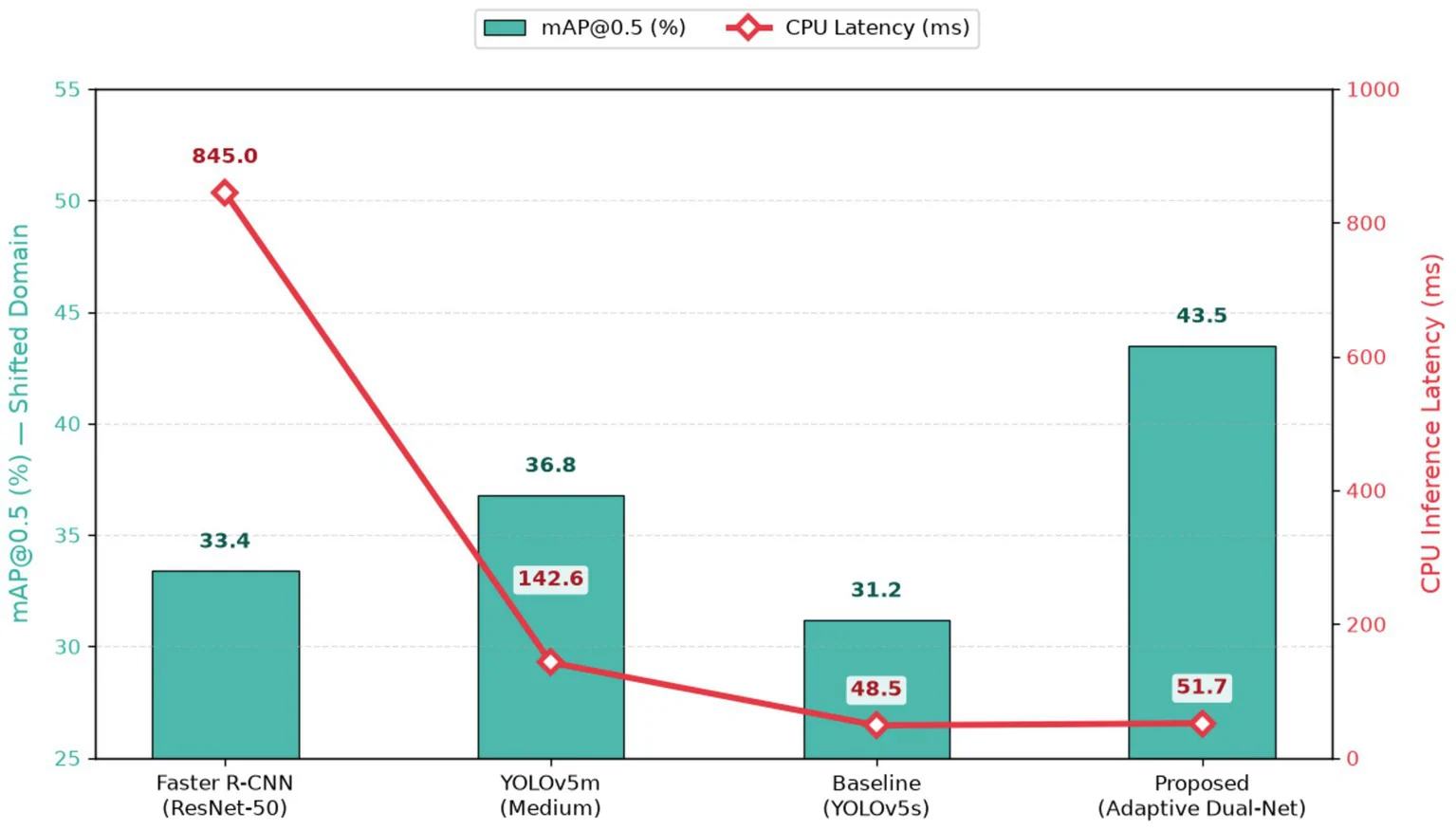
Comparative analysis of detection accuracy (mAP) and inference latency (Latency) on a dual-axis combination in a multi-network architecture under edge computing nodes.

Figure 7 shows the t-SNE topological manifold, which maps the feature guidance efficiency of the unsupervised continuous learning architecture in the high-dimensional latent space. When faced with dual-domain shift test sets of nighttime photometric distortion and extreme weather, Figure 7a shows that the various feature clusters of the base network (such as Car, Person, Rider, etc.) are more diffuse, with large-scale overlap of community boundaries. This confusion in the underlying semantic representation corresponds to its limited perceptual accuracy in nighttime and weather migration tasks (mAP@0.5 is only 31.2% and 34.6% respectively, and F1 scores are 0.335 and 0.375), confirming the adverse effect of spatial distribution shift on feature mapping. Observing the distribution state in Figure 7b, the proposed adaptive model, without manual annotation of the target domain, promotes the formation of a high intra-class aggregation and inter-class separation topological structure in the latent space. The sample points of each core category converge along the manifold surface, and the decision boundaries tend to be smooth and isolated. The spatial topology evolution shows that the dynamic pseudo-label and predictive distillation mechanism optimizes the gradient trajectory during backpropagation, guiding the network to fit the domain-invariant core feature manifold. Feature decoupling at the lower-level representation dimensions constructs a high signal-to-noise ratio classification boundary, supporting an enhancement in macroscopic perception metrics and improving the model’s mAP@0.5 to 43.5% and 47.1% in nighttime and weather migration tasks (achieving absolute performance gains of 12.3% and 12.5%, respectively). To further quantify the trade-off between spatial perception accuracy and underlying hardware computational overhead, the static metrics and dynamic inference performance of the multi-network architecture are summarized in Figure 8 and Table 3.

**Table 3:** Evaluation of computational complexity and inference time for different object detection architectures in a GPU-unaccelerated environment.

Detection architecture	Params (M)	FLOPs (G)	CPU Latency (ms)	FPS (frames per second)
Faster R-CNN (ResNet-50)	41.53	205.7	845.0	1.2
YOLOv5m (Medium Standard)	21.05	47.9	142.6	7.0
Baseline (YOLOv5s)	7.02	15.8	48.5	20.6
Proposed (Adaptive Dual-Net)	5.86	16.6	51.7	19.3

Based on effective alignment in the feature space, the dual Y-axis combination comparison diagram (Figure 8) and Table 3 further confirm the physical consistency between lightweight reconstruction and the system’s computational power boundary. Experimental data shows that although the traditional two-stage heavy network Faster R-CNN has a certain parameter capacity (41.53 M parameters), its single-frame inference latency is as high as 845.0 ms on x86 computing nodes without dedicated graphics accelerators, and the processing frame rate (1.2 FPS) falls short of practical engineering deployment requirements. The standard YOLOv5m reduces the latency to 142.6 ms (7.0 FPS) through a simplified architecture, while the YOLOv5s baseline network can reach the near real-time boundary of 48.5 ms (20.6 FPS). This model (Proposed) introduces depthwise separable convolutions to decouple and compress multiple channels, further reducing the static parameter count by 16.5% compared to the minimalist baseline, reaching 5.86 M. Although the complex cross-layer skip connections and element-wise fusion operations in BiFPN result in a reasonable slight increase in dynamic computation (FLOPs from 15.8–16.6 GFLOPs), its single-frame forward latency on a general-purpose CPU (51.7 ms) only increases by a mere 3.2 ms compared to the baseline network. The jump in mAP@0.5 from 31.2% to 43.5% on the left principal axis, together with the latency evolution trajectory represented by the right secondary axis, confirms that the significant gain in cross-domain accuracy offsets the slight computational cost. The system still maintains a processing frame rate of 19.3 FPS, meeting the minimum sampling threshold for dynamic scenarios, even on constrained physical hardware. This not only confirms the lightweight design intent but also demonstrates the physical feasibility of achieving low-power, long-term domain-adaptive deployment on constrained edge terminals.

## 6 Conclusion

This study constructs an adaptive visual diagnostic framework for complex scenes, integrating unsupervised pseudo-label learning and multi-scale feature extraction. By introducing a BiFPN node in the feature extraction stage, utilizing a dynamic threshold-constrained pseudo-label bidirectional recovery mechanism, and formulating a continuous learning constraint based on predictive distillation, the system facilitates perception adaptation. Experimental results indicate that on a standard CPU computing node, the system operates with a computational load of 16.6 GFLOPs and a processing frame rate of 19.3 FPS. In the illumination and meteorological domain migration tasks on the BDD100K benchmark, the target domain mAP@0.5 reaches 43.5% and 47.1%, and the mAP@0.5:0.95 reaches 22.1% and 24.5%, respectively. In domain incremental evolution tests, the architecture maintains the source domain mAP@0.5 at 52.8% after 10 iterations, showing a reduction in knowledge forgetting compared to parameter regularization (EWC) and response distillation (LwF) methods. This architecture provides a reference for optimizing feature extraction in open environments, addressing missed detections caused by domain shifts within edge computing limits. The system exhibits stable perception capabilities under lighting variations and weather conditions. When encountering unstructured sudden obstacles or long-tailed anomalies in continuous data streams, the existing pseudo-label recovery mechanism shows limitations in semantic recall, which may lead to missed detections of targets with local morphological changes. Future research will explore multimodal information fusion, such as depth sensing data, and large-scale model prior guidance to expand the applicability of diagnostic models in non-stationary open environments.

## Data Availability

The original contributions presented in the study are included in the article/supplementary material, further inquiries can be directed to the corresponding author/s.
